# Junctions in Jeopardy: the neuromuscular junction is a selective pathological target in Charcot-Marie-Tooth disease

**DOI:** 10.1007/s00335-026-10238-z

**Published:** 2026-05-22

**Authors:** Louisa Snape, James N. Sleigh

**Affiliations:** 1 Department of Neuromuscular Diseases and UCL Queen Square Motor Neuron Disease Centre, UCL Queen Square Institute of Neurology, University College London, London, UK; 2 UK Dementia Research Institute, University College London, London, UK

**Keywords:** Axon degeneration, CMT, Motor neuron, Peripheral neuropathy, Schwann cell, Synapse

## Abstract

Charcot-Marie-Tooth disease (CMT) is a genetic peripheral neuropathy arising from mutations in diverse genes that principally disrupt axons and Schwann cells. As the most distal synaptic interface of motor neurons, the neuromuscular junction (NMJ) represents a plausible but underexplored site at which such disruptions may converge to confer selective peripheral neuropathy. This review synthesises current evidence for NMJ involvement in CMT, focusing on mammalian systems, and evaluates how localised synaptic pathology relates to distal nerve dysfunction across genetic models. We outline the organisation of the mammalian NMJ and experimental approaches used to assess its dysregulation, emphasising the distinction between structural and functional denervation. Appraisal of NMJ abnormalities reported across axonal and demyelinating CMT models reveals evidence for impaired synaptic maturation, transmission and conduction failure, often prior to subsequent structural denervation and axonal degeneration. Emerging patterns indicate well-studied axonal subtypes show early, length-dependent synaptic dysfunction, whereas demyelinating forms often exhibit secondary NMJ destabilisation with ineffective axonal sprouting and reinnervation attempts. We also address methodological and interpretive considerations in NMJ studies, and consider the translational relevance of NMJ disruption as a functional readout of pathology and potential therapeutic target. Collectively, this review clarifies the NMJ as an informative, active and selective site of vulnerability in CMT, while demonstrating both the need and relevance for additional investigation in mammalian systems.

**Supplementary Information:**

The online version contains supplementary material available at 10.1007/s00335-026-10238-z.

## Introduction

### Charcot-Marie-Tooth disease

Charcot-Marie-Tooth disease (CMT) comprises a heterogeneous group of inherited peripheral neuropathies characterised by chronic, length-dependent motor and sensory dysfunction (Burns et al. 2026; Reilly et al. 2011). Clinically, CMT presents with slowly progressive distal muscle weakness and atrophy, sensory loss and skeletal deformities, with substantial variability in severity and onset age between genetic subtypes (Fridman & Saporta 2021). Diagnosis integrates clinical features with neurophysiology. Indeed, nerve conduction studies broadly stratify CMT into a) demyelinating forms (e.g. CMT1 or CMT4) with uniformly slowed nerve conduction velocities (NCVs), b) axonal forms (e.g. CMT2) with relatively preserved NCVs but reduced compound muscle action potential (CMAP) amplitudes, and c) intermediate forms (e.g. dominant intermediate CMT [DI-CMT]) with overlapping features (Pipis et al. 2019; Reilly et al. 2011). Notably, disability correlates poorly with slowing of NCVs but strongly with axonal degeneration and motor unit loss, establishing distal axon loss as a key driver of disease progression across both demyelinating and axonal CMT (Krajewski et al. 2000; Lawson et al. 2003; Lewis et al. 2003) – though the pathomechanisms underpinning selective peripheral neuropathy remain unresolved.

Genetically, CMT is one of the most heterogeneous neurological disorders, with pathogenic variants identified in over 100 genes; marked phenotypic overlap is apparent and inheritance from individual genes can be variable, complicating classification between distinct subtypes (DiVincenzo et al. 2014; Fridman & Saporta 2021; Morena et al. 2019). Next-generation sequencing has expanded gene discovery, but also blurred boundaries between CMT and the closely related motor- and sensory-predominant neuropathies, known as distal hereditary motor neuropathy (dHMN) and hereditary sensory neuropathy, respectively (Pipis et al. 2019; Rossor et al. 2024).

Mechanistically, CMT disrupts multiple cellular processes in peripheral nerves, including Schwann cell myelination, axonal transport, mitochondrial dynamics and proteostasis (Prior et al. 2017; Scherer & Wrabetz 2008; Suter & Scherer 2003). This convergence provides a framework to interrogate whether the most distal subcellular compartment of lower motor neurons, the neuromuscular junction (NMJ), represents an early and mechanistically informative site of vulnerability in CMT – as is the focus of this review.

### Introducing the NMJ in CMT

NMJ defects are increasingly demonstrated in diverse contexts – including ageing (Iyer et al. 2021; Moss et al. 2025), neuromuscular conditions such as amyotrophic lateral sclerosis (ALS) (Alhindi et al. 2022; Dadon-Nachum et al. 2011; Fischer et al. 2004) and spinal muscular atrophy (SMA) (Goulet et al. 2013; Murray et al. 2008), mitochondrial diseases (Braz et al. 2021; Lessard et al. 2024), inherited myopathies and muscular dystrophies (Ng & Ljubicic 2020; Rudolf et al. 2014), as well as myasthenic disorders, where pathology arises from primary synaptic dysfunction (Nicolau et al. 2019).

By contrast, the NMJ has historically received limited investigation in CMT, despite the central role of selective distal axonal degeneration – likely reflecting the traditional conceptualisation of peripheral neuropathy as being a Schwann cell or axonal disorder (Moss et al. 2025; Soh et al. 2020). Nevertheless, the NMJ has become increasingly well studied in CMT models; preliminary cross-field syntheses recognise that multiple CMT mouse models exhibit early NMJ degeneration (Pisciotta & Pareyson 2023; Scherrer et al. 2025; Vendredy et al. 2025). Direct NMJ abnormalities have also been reported in CMT patients with mutations in genes such as *GARS1*, *DYNC1H1*, *DNM2* and *SYT2* (McMacken et al. 2023). Most notably, the ESTABLISH study identified impaired NMJ transmission as a quantifiable disease feature in both CMT1 and CMT2 patients, with increased jitter and neurotransmission failure correlating with clinical severity (Grønnebæk et al. 2024).

Common clinical electrophysiology measures, such as CMAP amplitudes, provide indirect evidence of NMJ dysfunction based on muscle function, but integrate multiple pre and postsynaptic conductive and architectural variables of the motor system, highlighting the need for further NMJ-specific diagnostic and functional assessments in CMT patients and models (Lewis et al. 2003; Rodriguez-Falces & Place 2018).

### Dying-back versus localised NMJ pathology

CMT is commonly described as a distal, length-dependent or “dying-back” neuropathy, reflecting its distal-predominant clinical features combined with consistent electrophysiological and pathological evidence of axon loss across subtypes (Bienfait et al. 2007; Morant et al. 2021; Prior et al. 2017; Rossor et al. 2016; Scherer 2006; Shackleford et al. 2022). However, the mechanistic basis of this distal vulnerability remains poorly defined, and “dying-back” has largely functioned as a descriptive shorthand rather than a mechanistic explanation of disease.

This contrasts with ALS, where the term originated to describe degeneration initiated at the NMJ or distal axon, which then progresses retrogradely towards motor neuron soma (Dadon-Nachum et al. 2011; Fischer et al. 2004). In ALS, distal axon pathology is closely linked to subsequent motor neuron death, whereas in CMT, axonal degeneration typically occurs without neuron loss (Bruijn et al. 2004; Nave et al. 2007). Extensive dissection across ALS models has further shown that NMJ pathology can arise through multiple, non-linear mechanisms, varying in timing, muscle fibre type susceptibility, regenerative capacity, and spatial progression (Alhindi et al. 2022; Fischer et al. 2004; Frey et al. 2000; Martineau et al. 2018; Moloney et al. 2014; Schaefer et al. 2005). These studies emphasise distal dysfunction as an active, heterogeneous process rather than simply a passive consequence of axon length.

Such distinctions are especially relevant given the genetic heterogeneity of CMT, where mutations affecting axon or Schwann cell function may converge on distal failure through diverse pathways (McCray & Scherer 2021; Reilly et al. 2011). Moreover, not all genes conform to an exclusively length-dependent paradigm of pathology, since a number of subtypes and even mutations can show predominant upper-limb severity (e.g. *GARS*, *REEP1* and *BSCL2*) or craniofacial involvement (e.g. *TRIM2*, *AIFM1, SH3TC2*) (Fridman & Saporta 2021; Magri et al. 2020; McMacken et al. 2023; Pipis et al. 2019). Together, these observations position the NMJ as an underexplored yet potentially informative site of pathology in CMT, while cautioning against uniform treatment of distal phenotypes, and underscoring the need for deeper investigation into molecular mechanisms driving peripheral neuropathy.

In this review, we will therefore examine different types of NMJ pathology, reveal emergent patterns of NMJ dysregulation in CMT models through providing a comprehensive summary of axonal and then demyelinating neuropathies, and conclude by identifying existing gaps that currently preclude a more substantial and integrated interpretation of the causes and/or consequences of NMJ dysfunction in peripheral neuropathy.

## The NMJ in health and disease

### NMJ components

**Fig. 1:**
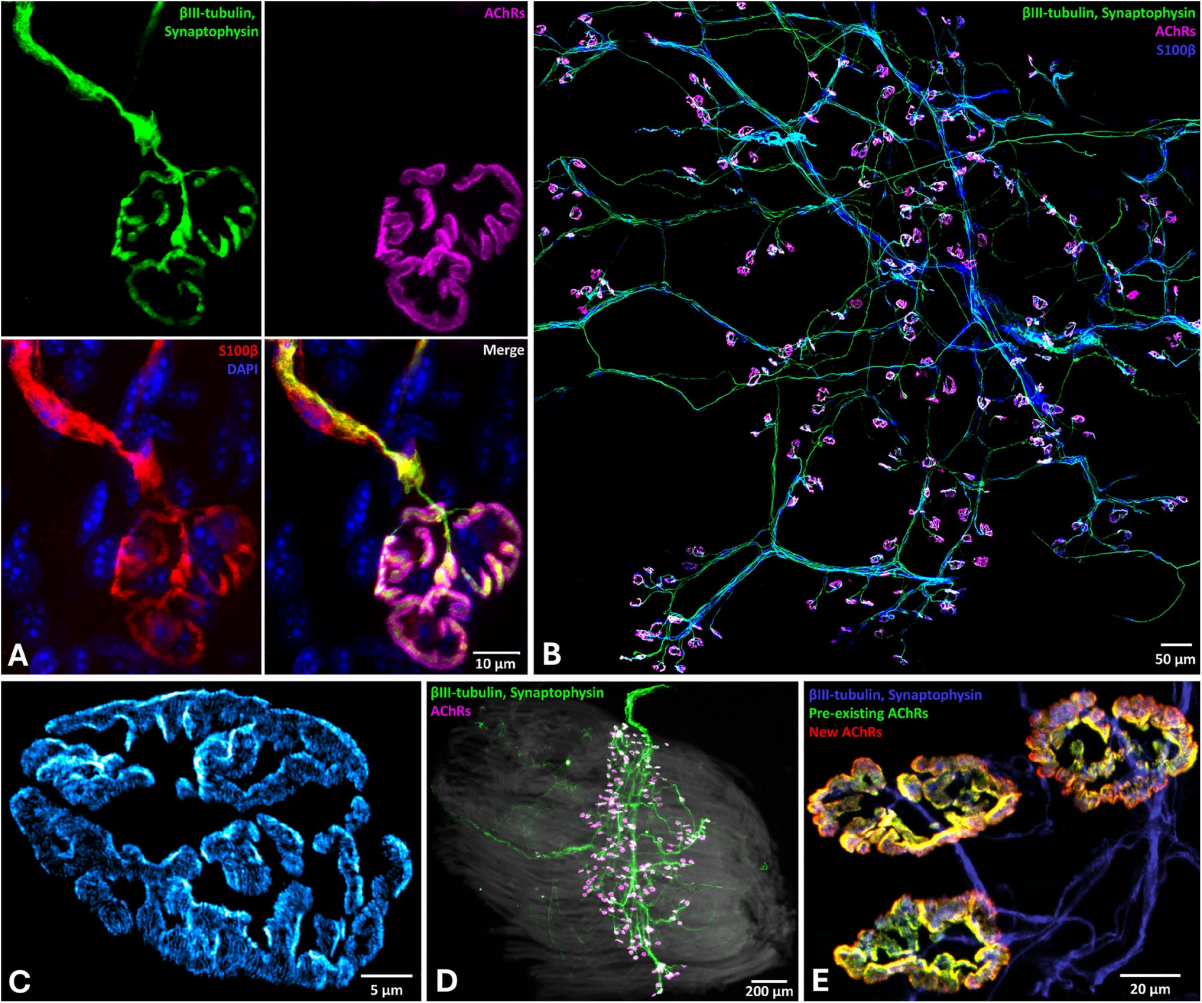
Visualising the NMJ. **A** The multicellular interactions present at individual NMJs are depicted via a 63 × maximum intensity confocal micrograph projection. Motor neurons are visualised using antibodies to detect microtubules (βIII-tubulin, green) and presynaptic terminals (synaptophysin, green), while postsynaptic terminals are labelled with αBTX (magenta) to detect AChRs. Axonal and terminal myelinating Schwann cells are detected with S100β (red) and cell nuclei with DAPI (blue). Scale bar = 10 µm. **B** A 20 × maximum intensity confocal micrograph projection shows the network of motor neurons (βIII-tubulin and synaptophysin, green) and their supporting myelinating Schwann cells (S100β, blue) innervating postsynaptic endplates (αBTX, magenta) on skeletal muscle fibres to form NMJs. Scale bar = 50 µm. **C** Semi-super resolution imaging via SoRa spinning disk microscopy at 60 × with deconvolution processing reveals the precision of postsynaptic organisation and patterned alignment of AChRs (αBTX, blue) along banded junctional folds of the muscle membrane. Scale bar = 5 µm. **D** A 2.5 × widefield image of a whole-mount muscle preparation displays the entire motor neuron innervation network and all NMJs present along skeletal muscle fibres in an individual mouse lumbrical muscle, visualising motor neuron microtubules (βIII-tubulin, green) and presynaptic terminals (synaptophysin, green), and postsynaptic terminals via α-BTX (magenta) to detect AChRs. Scale bar = 200 µm. **E** Dynamic postsynaptic NMJ maintenance and turnover is illustrated via pulse-chase labelling with two separate αBTX labels applied at two distinct time points to demarcate different subpopulations of AChRs – pre-existing (green) and subsequently newly synthesised (red). Innervating motor neurons (βIII-tubulin and synaptophysin, blue) are also labelled. Scale bar = 20 µm

**Fig. 2:**
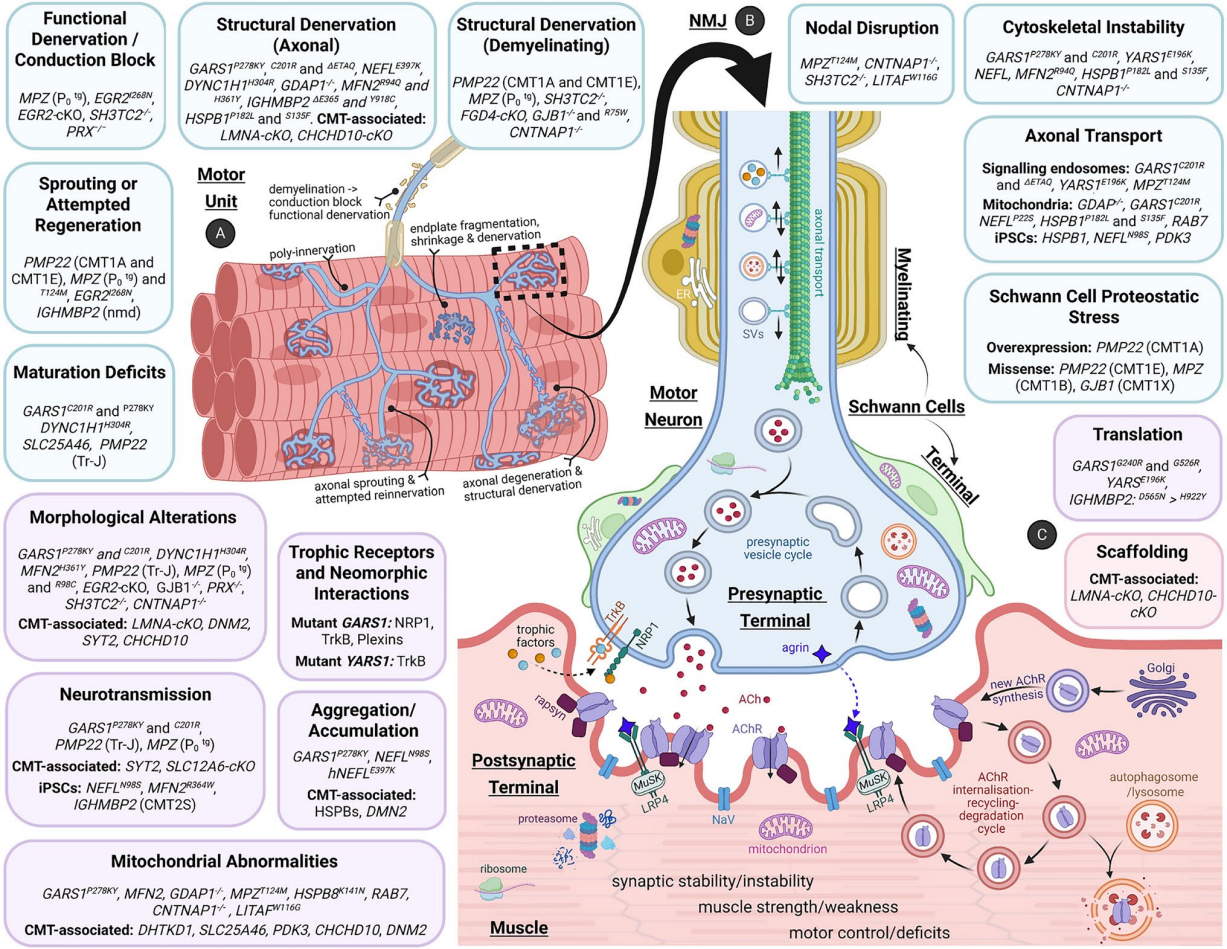
System and cellular-level pathology at the NMJ in CMT models. **A** Motor unit-level manifestations of NMJ pathology. A motor neuron innervating skeletal muscle fibres illustrates healthy, alongside dysfunctional, NMJs at the motor unit level, including distal presynaptic poly-innervation, structural axonal degeneration and denervation, conduction block-associated functional denervation, and axonal sprouting with attempted reinnervation, alongside postsynaptic fragmentation and motor endplate dysmorphology. **B** Cellular and subcellular organisation of the mammalian NMJ. An individual NMJ within the motor unit depicts the tripartite configuration of the motor neuron presynaptic terminal (maintained by cytoskeletal integrity, diverse cargo delivery, and local proteostatic turnover to sustain neurotransmitter release); terminal/myelinating Schwann cells; and the postsynaptic muscle endplate (enriched in AChRs and associated scaffolding proteins, where dynamic trophic signalling, receptor recycling, and organelle positioning coordinate synaptic stability and long-term motor output). **C** Components and molecular pathways implicated in NMJ dysfunction in CMT. Distinct patterns of pathology seen at the NMJ are mapped to canonical CMT and CMT-associated gene variants and models, organised by primary sites of action: presynaptic (blue – e.g. denervation, axonal transport, nodal disruption), shared pre-/postsynaptic or multi-compartmental (purple – e.g. morphological and mitochondrial abnormalities, neurotransmission, aggregation, translation defects), and postsynaptic (red – e.g. scaffolding and receptor protein dysfunction). These associations are also summarised in Supplementary Table 1

The NMJ is a highly specialised peripheral synapse composed of motor axons, skeletal muscles, Schwann cells (Fig. 1A-B, Fig. 2A), and extracellular matrix (ECM) that is under tight regulation via bidirectional signalling and complex protein scaffolds (Fig. 2B) (Grinnell 1995; Rodríguez Cruz et al. 2020). Because CMT-causing genes disrupt both axon and Schwann cell functions, the NMJ represents a key site where disease-relevant cell types converge, rendering neuromuscular connectivity particularly vulnerable in neuropathy.

Structurally, the presynaptic component comprises motor axon terminals forming synaptic boutons (*i.e.* terminal enlargements) enriched with acetylcholine (ACh)-filled synaptic vesicles (SVs) and active zone machinery for rapid and efficient neurotransmitter release (Fig. 2B) (Desaki & Uehara 1981; Südhof 2012). Vesicle docking, fusion, and recycling depend on voltage-gated calcium channels, SNARE complexes, and scaffolding proteins (Baker & Hughson 2016; Rizzoli 2014), where disruption can drive primary synaptopathies, for instance Lambert-Eaton myasthenic syndrome (Titulaer et al. 2011).

The synaptic cleft contains a specialised basal lamina that aligns pre and postsynaptic elements and provides essential signalling and structural functions. Composed of laminins, collagens, and proteoglycans, the ECM anchors synaptic components and localises acetylcholinesterase to ensure quick termination of neurotransmission and maintenance of temporal fidelity (Ohno et al. 1998; Sanes 2003; Shi et al. 2012).

The postsynaptic muscle membrane at the NMJ is defined by deep junctional folds that expand surface area and segregate signalling domains (Slater 2017). Pentameric nicotinic acetylcholine receptors (AChRs) cluster densely at fold crests (Fig. 1C), precisely aligned with presynaptic active zones, whereas voltage-gated sodium channels (NaVs) concentrate at fold bases to ensure efficient action potential initiation (Fig. 2B) (Liu et al. 2019; Wang et al. 2017; York & Zheng 2017). Postnatally, NMJ maturation involves a switch from γ- to ε-containing AChRs, conferring faster channel kinetics and higher conductance, together with a transition from simple plaque-like clusters to complex pretzel-shaped architectures to orchestrate coordinated functional and morphological refinement (Marques et al. 2000; Shi et al. 2012; Witzemann et al. 1987). Organisation and long-term stability of these structures depend on local protein turnover and bidirectional agrin-LRP4-MuSK signalling, with rapsyn anchoring AChRs to both the cytoskeleton and dystrophin-associated complexes (Fig. 2B) (Barik et al. 2014; DeChiara et al. 1996; Ham et al. 2025; McMahan 1990; B. Zhang et al. 2008). The postsynaptic endplate is a shared site of vulnerability across neuromuscular disorders; this is exemplified by diverse myopathies, where primary muscle fibre defects lead to disruption of postsynaptic organisation, and by the most common disorder of neuromuscular transmission, myasthenia gravis (MG), in which autoantibodies directly target AChRs (Engel et al. 2015; Ogasawara & Nishino 2023).

Terminal Schwann cells (tSCs) cap the NMJ (Fig. 1A, Fig. 2B) and are essential for synaptic stability, plasticity, and regeneration. Beyond trophic support, tSCs actively sense neurotransmitter release and modulate synaptic efficacy (Santosa et al. 2018). Following denervation, Schwann cells extensively reprogramme into a repair phenotype marked by c-JUN activation, suppression of myelin genes, secretion of neurotrophic factors, and morphological sprouting to form Büngner bands that guide regenerating axons to vacant endplates (Arthur-Farraj & Coleman 2021; Gomez-Sanchez et al. 2017; Jessen & Arthur-Farraj 2019; Son & Thompson 1995). Schwann cells also support axonal metabolism independently of myelination and act as local immunomodulators during degeneration (Belančić et al. 2025; Reed et al. 2022). Importantly, the peripheral nervous system contains heterogeneous Schwann cell populations – including myelinating, terminal, and satellite cells – many of which represent primary or contributory pathological targets in CMT and related motor neuron disorders, underscoring glial function as a critical determinant of NMJ homeostasis (Court et al., 2008b; Reed et al. 2022).

### Assessing NMJ dysregulation

#### NMJ visualisation

At the cellular level, NMJ structure and integrity are most commonly assessed by immunolabelling pre and postsynaptic components in dissected muscles. Postsynaptic AChRs are typically labelled with α-bungarotoxin (αBTX), combined with presynaptic markers, such as synaptophysin or synaptic vesicle protein 2, and axonal cytoskeletal markers, including βIII-tubulin or neurofilament in rodents and zebrafish (Hsieh & Chen 2024; Simkin et al. 2025; Sleigh et al. 2014a), or anti-horseradish peroxidase antibodies in *Drosophila melanogaster* larvae (Menon et al. 2013). Schwann cells and muscle fibres can additionally be visualised using markers such as S100β and phalloidin, respectively (Fig. 1A) (Castro et al. 2020; Vettori et al. 2011). Whole-mount (Fig. 1B, 1D), sectioned or teased fibre muscle preparations enable microscopy and quantitative analysis of NMJ innervation, alignment, and morphology across motor units. Higher-resolution approaches, including super-resolution, expansion or electron microscopy, can resolve nanoscale synaptic and organelle organisation (Fig. 1C) (Badawi & Nishimune 2020; Modla et al. 2010; Ramadan et al. 2025). Longitudinal in vivo imaging using fluorescent reporter lines further captures NMJ remodelling over time, highlighting strong dynamic plasticity of the synapse (Lichtman & Sanes 2003; Negro et al. 2022; Turney et al. 2012; Walsh & Lichtman 2003).

#### Abnormal innervation

During development, muscle fibres are transiently innervated by multiple axons (*i.e.* poly-innervation (Fig. 2A)) before the process of synapse elimination results in localised and activity-dependent removal of supernumerary axons to generate singly innervated, stable NMJs (Brown et al. 1976; Lømo & Rosenthal 1972; Sanes & Lichtman 1999; Walsh & Lichtman 2003). As synapse elimination is tightly coupled to nerve-muscle electrical activity, defects in neurotransmission can precede structural withdrawal during development and disease (Brown et al. 1976; Gillingwater et al. 2002). Non-normal innervation at the NMJ also includes structural denervation (Fig. 2A) – a feature shared across ageing, sarcopenia, muscular dystrophies, ALS, SMA and other neuromuscular disorders (Alhindi et al. 2022; Iyer et al. 2021; Rudolf et al. 2014; Villalón et al. 2018). Denervation reflects physical loss of presynaptic contact with the postsynaptic endplate, typically marked by motor terminal retraction and axonal degeneration (X. Huang et al. 2022). Early changes, defined largely through nerve injury models, include redistribution of AChRs to extrajunctional sites, reduced membrane excitability, and re-expression of the immature γ AChR subunit (Hartzell & Fambrough 1972; Sanes & Lichtman 1999; Steinbach 1981; Witzemann et al. 1987). Sustained denervation results in muscle inactivity, atrophy and fibrosis, and is accompanied by pronounced NMJ remodelling, including reduced endplate size and postsynaptic fragmentation (Bermedo-García et al. 2022; Marques et al. 2000; Yin et al. 2019). Axon loss can also engage Wallerian degeneration, an active self-destruction programme mediated by *SARM1*-dependent NAD⁺ depletion and cytoskeletal collapse (Coleman & Hoke 2020; K. Zhang et al. 2021c). In CMT, axon degeneration is observed in axonal but also demyelinating CMT, where Schwann cell dysfunction is increasingly additionally recognised as an active driver of secondary axonal degeneration rather than a passive consequence of myelin loss (Moss et al. 2021; Nave et al. 2007).

Contrastingly, states of *functional denervation* can also occur at the NMJ (Fig. 2A) – whereby motor neurons still faithfully occupy the endplate terminal but fail to effectively activate neurotransmitter release due to impaired impulse propagation or conduction block, leading to compromised neurotransmission through an otherwise structurally intact axon (Kaji 2003). Mechanistically, functional denervation – such as conduction block – can arise when myelin abnormalities disrupt membrane capacitance and saltatory conduction, destabilising internodal ion channel organisation and synaptic excitability (Kiernan et al. 2002; Scherer & Wrabetz 2008). Importantly, functional denervation is not passive; reduced muscle activity induces ACh hypersensitivity through extrajunctional receptor upregulation along muscle fibres, which can in turn trigger transient axonal sprouting from neighbouring terminals (Brown & Holland 1979; Lomo & Westgaard 1975; Pasino et al. 1996; Tam et al. 2001). Such sprouting is reversible and persists only while conduction failure remains, distinguishing it from stable compensatory reinnervation (Tam et al. 2001; Tam & Gordon 2003). Repeated cycles of denervation and reinnervation thus further generate immature sprouts with unstable conduction, reinforcing functional transmission deficits despite preserved morphology (Verma et al. 2022). Functional denervation therefore represents an underappreciated mechanism of NMJ dysfunction particularly relevant to CMT, and one likely overlooked due to the apparently intact appearance of axons.

These repeated cycles of denervation and attempted reinnervation alongside axonal sprouting (Fig. 2A) are observed across ageing and multiple neuromuscular diseases, and challenge the assumption that regenerative signatures are inherently beneficial (Aare et al. 2016; Ang et al. 2010; Comley et al. 2022; Hepple & Rice 2016). In *SOD1* ALS models, in vivo imaging demonstrates asynchronous dismantling of distal motor units alongside concurrent axonal sprouting and new synapse formation weeks before overt axon loss, providing evidence for the dynamic “dying-back” process (Fischer et al. 2004; Frey et al. 2000; Martineau et al. 2018; Schaefer et al. 2005). However, axonal sprouting is often transient and poorly targeted, constrained by temporally declining Schwann cell support, and frequently fails to restore effective transmission (Gaudet et al. 2011; Gordon 2020; Sakuma et al. 2016). Thus, regenerative remodelling may reflect persistent instability rather than durable functional recovery.

#### Functional neurotransmission

Functionally, neuromuscular transmission is conventionally safeguarded by a substantial safety factor. In rodents, each motor action potential triggers a quantal release of ACh far exceeding the threshold required to elicit a muscle action potential (Wood & R. Slater, 2001). This redundancy allows NMJs to tolerate partial denervation, reduced quantal content, or structural abnormalities while retaining transmission. A clear example concerns postsynaptic endplate fragmentation (Fig. 2A), widely reported in ageing, dystrophy, ALS, SMA, and experimental denervation (Rudolf et al. 2014; Valdez et al. 2010; Villalón et al. 2018). Following nerve injury, fragmentation can occur alongside preserved neurotransmission (Nagel et al. 1990; Slater 2020), where detailed morphometric analyses distinguish stable, smooth fragmentation compatible with functional recovery from irregular, blurred fragmentation associated with persistent denervation and declining transmission (Bermedo-García et al. 2022). Indeed, diseased NMJs with reduced endplate size, partial denervation, or neurofilament accumulation are additionally shown to retain effective transmission for prolonged periods, with functional deficits emerging only gradually as the safety margin erodes (Alhindi et al. 2022; Gillingwater et al. 2002; Kong et al. 2009). Consequently, morphological hallmarks of NMJ remodelling or attempted regeneration may reflect attempted compensation, and do not necessarily equate to functional failure or inevitable further degeneration. These features highlight the critical importance of assessing synaptic transmission alongside structure when evaluating NMJ health.

Beyond visual methods, NMJs can also be interrogated using electrophysiological approaches to directly quantify synaptic transmission. For instance, intracellular recordings from muscle fibres measure miniature and evoked endplate potentials or currents (mEPPs/mEPCs; EPPs/EPCs), enabling assessment of quantal size or content and release frequency as key determinants of presynaptic efficacy and postsynaptic responsiveness (Scurry et al. 2016; Spaulding et al. 2016; Yin et al. 2004). Repetitive nerve stimulation or single-fibre electromyography (EMG) evaluate release probability and vesicle recycling, with *failure analysis* detecting high-frequency transmission instability and reduced safety margins (Amaral et al. 2011; Court et al. 2008a; Gillingwater et al. 2002; McMacken et al. 2023; Meekins et al. 2007). Together, these approaches can sensitively distinguish deficits in functional competence irrespective of structural appearance.

Additionally, motor neuron hyperexcitability is increasingly recognised as a shared phenotype across neuromuscular and neurodegenerative disorders, most prominently in ALS, through observations across presymptomatic *SOD1*^G93A^ mice (Kuo et al. 2004; Ruegsegger et al. 2016; Tortarolo et al. 2006) and human induced pluripotent stem cell (iPSC)-derived motor neurons modelling *SOD1*, *FUS*, *C9orf72* and TDP-43 dysfunction (Joseph et al. 2025; Keuss et al. 2024; Wainger et al. 2014). Whether hyperexcitability is compensatory, pathogenic, or context-dependent remains unclear (Gunes et al. 2020; Leroy & Zytnicki 2015; Odierna et al. 2024; Saxena et al. 2013). Therefore, resolving such contribution to NMJ dysfunction will also require electrophysiological analysis integrated with structural assessment across defined disease stages.

#### Postsynaptic alterations

The possibility for dissociation between structure and function substantially complicates interpretation of NMJ pathology. Moreover, morphological assessment is further confounded by qualitative variability – for instance, how fragmented must an endplate appear in order to be classified as “fragmented”? To address this subjectivity, standardised, computationally driven platforms increasingly enable formalised, high-throughput quantification of NMJ organisation and innervation. Tools such as NMJ-morph, and its automated extension aNMJ-morph, extract ~ 20 pre and postsynaptic parameters from two-dimensional (2D) projections, while NMJ-Analyser incorporates automated thresholding, cross-experiment normalisation and machine learning-based classification of innervation using three-dimensional (3D) reconstructions (Jones et al. 2016; Mejia Maza et al. 2021; Minty et al. 2020). Comparable frameworks have also been developed across zebrafish, invertebrate models and human neuromuscular co-culture systems, converging on shared objective and quantitative descriptors, including receptor distribution, synaptic occupancy, fragmentation and stability indices (Badu-Mensah et al. 2022; Lescouzères et al. 2022; Singh et al. 2023; Soh et al. 2020).

Beyond gross morphology, altered AChR density and turnover add an important layer of complexity to postsynaptic NMJ maintenance (Fig. 1E). In intact adult muscle, AChRs are metabolically stable, with an average half-life of ~ 10-14 days across receptor pools (Akaaboune et al. 1999; Martinez-Pena Y Valenzuela & Akaaboune 2021). This stability is sustained by an activity-dependent dynamic equilibrium, in which coordinated endocytosis, recycling and vesicular insertion or degradation of receptors collectively determine postsynaptic AChR density (Fig. 2B) (E. Bruneau et al. 2005; Khan et al. 2014; Marchand et al. 2000; Rudolf & Straka 2019). Disruption of receptor cycling or the spatial precision of insertion or recycling can thus alter synaptic stability and potentially contribute to endplate fragmentation or ectopic AChR expression (Brenner & Akaaboune 2014; E. Bruneau et al. 2005; Rudolf et al. 2014).

These processes are proposed to be regulated by second-messenger pathways, with protein kinase A promoting receptor stabilisation and recycling, protein kinase C accelerating internalisation, and calcium/calmodulin-dependent protein kinase II coupling calcium influx to activity-dependent cycling, in association with receptor anchoring proteins such as rapsyn and myosin (Choi et al. 2012; Nelson et al. 2003; Röder et al. 2010; Valenzuela et al. 2010, 2013). Downstream, ubiquitin- and autophagy-mediated pathways, including MuRF1/TRIM63 and p62, link AChR loss to muscle atrophy programmes (Khan et al. 2014; Wild et al. 2016).

Much of our understanding of AChR turnover derives from classic models of complete, acute denervation, which severs all neuromuscular connectivity, emphasising rapid receptor loss, shortened half-life and synaptic destabilisation (E. G. Bruneau & Akaaboune 2006; Hartzell & Fambrough 1972; Levitt & Salpeter 1981; Röder et al. 2010; Stanley & Drachman 1981). However, factors beyond complete functional denervation appear to affect postsynaptic organisation, since direct muscle stimulation preserves AChR stability even in denervated muscle (Brenner & Rudin 1989; Rotzler & Brenner 1990). Additionally, altered AChR turnover can also arise under conditions of partially preserved but dysfunctional innervation alongside alternate hallmarks of distal pathology, including *SOD1* ALS models showing increased receptor turnover and fragmentation independently of nerve transection (Dobrowolny et al. 2018).

Together, these observations underscore the need for studies that directly examine physiological denervation alongside AChR turnover and postsynaptic instability to capture active, but perhaps maladaptive, postsynaptic remodelling that may drive NMJ dysfunction.

#### Chronic NMJ vulnerability

It is potentially problematic for understanding neuropathy that much of our knowledge of NMJ disruption derives from artificial injury paradigms, such as proximal nerve transection or pharmacological blockade, given that CMT is a slowly progressive, chronic disorder (Bermedo-García et al. 2022; E. G. Bruneau & Akaaboune 2006; Menorca et al. 2013; Sakuma et al. 2016). This reliance partly reflects limited access to longitudinal and gene expression data following chronic nerve injury (Cavalcanti et al. 2009). Acute injury models are more accessible, but impose abrupt and complete loss of upstream neural input, activating Wallerian degeneration, macrophage infiltration and robust repair programmes that contrast with the length-dependent, distal “dying-back” mechanisms proposed to underlie CMT pathogenesis (Arthur-Farraj & Coleman 2021; Gaudet et al. 2011; Prior et al. 2017). Although chronic injury can elicit Schwann cell responses overlapping with those observed after acute injury (Atanasoski et al. 2006; Gupta et al. 2006), the temporal sequence and bidirectional signalling processes operating at the NMJ in chronic disease remain incompletely defined (Iyer et al. 2021; Rudolf et al. 2014). In this context, models of ageing, sarcopenia and ALS may better capture chronic NMJ vulnerability, revealing gradual functional compensation and slow terminal withdrawal driven by cumulative local stressors rather than acute axonal severing (Rudolf et al. 2014; Schaefer et al. 2005; Yin et al. 2004). Jointly, these observations underscore the need for caution when extrapolating mechanisms from acute injury paradigms and highlight the importance of further studies aimed at elucidating the precise molecular alterations occurring at the NMJ in CMT.

Overall, the NMJ emerges as a key site of vulnerability precisely because it integrates complex multicellular local signals from neurons, Schwann cells and muscle fibres, and is located at the terminals of long, thin axons far from the nucleus. Hallmarks such as denervation, reinnervation, fragmentation, and receptor turnover cannot be interpreted in isolation. Instead, they must be understood within the framework of safety factor dynamics, bidirectional signalling, and temporal progression. Overreliance on acute injury models has shaped prevailing paradigms, yet creates risks of obscuring the slower, local and context-dependent nature of NMJ pathology in ageing and disease. Valuable NMJ investigation thus prioritises diverse, longitudinal approaches that capture the continuum of NMJ states – bridging structure, function, and signalling to most accurately reflect and resolve physiological disease processes.

## NMJ pathology in CMT

### Axonal CMT

Axonal CMT arises from primary motor and sensory neuron dysfunction, with relative preservation of myelin early in disease, and progressive, length-dependent axonal degeneration as a defining feature. CMT2 subtypes are most commonly autosomal dominant, affecting genes encompassing axonal transport, cytoskeletal organisation, mitochondrial dynamics, RNA processing and synaptic maintenance (Reilly et al. 2011; Rossor et al. 2024). These pathways are essential for sustaining long axons, rendering distal compartments particularly vulnerable to disruptions in energy supply, cargo trafficking and local protein homeostasis (McCray & Scherer 2021). Consequently, the NMJ represents a logical and more frequently interrogated site of early pathology in axonal CMT than demyelinating CMT, with evidence supporting that synaptic dysfunction and impaired maintenance can arise alongside and often before overt axonal degeneration (Scherrer et al. 2025).

#### Aminoacyl-tRNA synthetases

Among the diverse genetic causes of CMT, the aminoacyl-tRNA synthetases (aaRSs) have emerged as the largest CMT-linked gene family (Rhymes & Sleigh 2026; Wei et al. 2019). aaRS genes are ubiquitously expressed, and play crucial roles in protein synthesis and translation through charging amino acid isoacceptors to cognate tRNAs (Burgess & Storkebaum 2023).

Mutations in these enzymes lead to dominant forms of CMT, producing a spectrum ranging from severe, early-onset neuropathy to milder, late-onset presentations (Wei et al. 2019). Mechanisms underlying selective peripheral nerve vulnerability remain incompletely resolved, but are unlikely to reflect sole loss of canonical aminoacylation function, as homozygous recessive *aaRS* mutations that impair enzymatic activity instead cause severe developmental multisystem disorders without peripheral neuropathy (Meyer-Schuman & Antonellis 2017). Although some CMT-aaRS variants reduce and even abolish enzymatic activity, accumulating evidence supports predominant toxic gain-of-function effects driven by conformational relaxation and aberrant neomorphic interactions (Kalotay et al. 2023; H. Zhang et al. 2021a).

Mutations in at least seven aaRS genes have been linked to CMT, beginning with glycyl-tRNA synthetase (*GARS1*) in CMT2D (Antonellis et al. 2003), followed by *YARS1* (DI-CMTC) (Jordanova et al. 2006), *AARS1* (CMT2N) (Latour et al. 2010), *HARS1* (CMT2W) (Vester et al. 2013), *WARS1* (dHMN9) (Tsai et al. 2017), *SARS1* (J. He et al. 2023), and *NARS1* (Beijer et al. 2024). *GARS1* and *YARS1* are the most well studied, providing some of the most comprehensive evidence to support impaired NMJ function in CMT, whereas the NMJ remains largely unexplored in all other aaRS-linked neuropathies.

#### GARS1

*GARS1* was the first aaRS gene linked to CMT, with pathogenic variants causing autosomal dominant CMT2D and motor-predominant dHMN-V (Antonellis et al. 2003). The *Gars*^*P278KY/*+^ mouse (also known as *Gars*^*Nmf249*/+^) arose spontaneously and recapitulates distal muscle weakness and axonal neuropathy, while revealing early NMJ pathology characterised by structural denervation and abnormal endplate morphology in hindlimb muscles without motor neuron loss (Seburn et al. 2006). A milder, mutagen-induced allele, *Gars*^*C201R/*+^, similarly demonstrates NMJ denervation and functional impairment while preserving lifespan and motor neuron number (Achilli et al. 2009); thus, both mutants accurately reflect a distal neuropathology. Longitudinal analyses established that NMJ degeneration is temporally and spatially restricted; in distal lumbrical muscles, defects in NMJ maturation, including persistent poly-innervation and delayed γ-to-ε AChR subtype switching, precede denervation, while proximal muscles such as the transversus abdominis are largely spared (Sleigh et al., 2014b). At postnatal day 15 (P15) to P16, NMJs in *Gars*^*C201R/*+^ mice are fully innervated, but progressive loss of synaptic occupancy emerges by one and three months, indicating degeneration rather than failed synaptogenesis (Sleigh et al. 2014b). These changes scale with allelic severity and mirror length-dependent clinical vulnerability. A broader survey across five whole-mounted muscles confirmed graded denervation, with distal hindlimb muscles exhibiting up to ~ 20% complete denervation by three months, while proximal muscles remain intact (Sleigh et al. 2020).

Functional studies showed that structural abnormalities are accompanied by early presynaptic failure. Quantal analysis in *Gars*^*P278KY/*+^ and *Gars*^*C201R/*+^ mice revealed reduced spontaneous release frequency, decreased evoked endplate currents and reduced quantal content, consistent with pleiotropic weakening of presynaptic function rather than a single dominant defect (Spaulding et al. 2016). Pharmacological enhancement of postsynaptic signalling with physostigmine, but not presynaptic function with 3,4-diaminopyridine, partially rescued neuromuscular performance and NMJ innervation, establishing the NMJ as an early and mechanistically informative site of pathology in *GARS1*-associated neuropathy. Later work further substantiated early NMJ involvement while delineating convergent molecular mechanisms that can be grouped into three dominant pathogenic streams. First, *GARS1* mutations confer a toxic gain-of-function through neomorphic protein interactions absent in wild-type glycyl-tRNA synthetase (GlyRS) that require conformational opening (Grice et al. 2015; W. He et al. 2015; Motley et al. 2011). These changes permit aberrant extracellular interactions with neuronal receptors, including neuropilin-1 (NRP1) and tropomyosin receptor kinase (Trk) family members, resulting in an accumulation of muscle-derived mutant GlyRS at the NMJ prior to degeneration in *Drosophila* models (Grice et al. 2015). Mutant GlyRS competitively inhibits vascular endothelial growth factor (VEGF) binding to NRP1 (W. He et al. 2015; Sleigh et al. 2017b) and interacts with TrkA, TrkB and TrkC, disrupting brain derived neurotrophic factor (BDNF) signalling (Sleigh et al. 2017a ; Sleigh et al. 2023). Additional interactions occur with semaphorin receptors, required for axon guidance and synaptic refinement, providing a mechanistic basis for persistent poly-innervation via disrupted trans-synaptic signalling at the NMJ (Grice et al. 2018). Intriguingly, BDNF functions in activity-dependent calcium channel clustering and neurotransmission, which regulate synaptic activity and elimination of redundant inputs, promoting poly-innervation (Dombert et al. 2017; Je et al. 2012; Mantilla et al. 2014; Nadal et al. 2016). Conversely, TrkB sequestration by mutant GlyRS may redirect BDNF toward p75^NTR^-mediated degenerative signalling and reduce TrkB activity, a state known to impair NMJ maintenance (Gonzalez et al. 1999; Pathak et al. 2021). Muscle-specific differences in TrkB and BDNF availability may therefore contribute to both aberrant synaptic maturation and selective denervation in CMT; indeed, TrkB levels in wild-type muscles closely correlate with susceptibility to denervation in CMT2D mice (Sleigh et al. 2023). Collectively, these findings position circulating mutant GlyRS as a pathological effector acting non-cell autonomously at the nerve-muscle interface to disrupt synaptic connectivity and activity.

A second major pathogenic theme involves impaired translation and chronic activation of the integrated stress response (ISR). In *Drosophila*, mutant GlyRS induces motor neuron-selective suppression of nascent protein synthesis rather than simple loss of aminoacylation, preceding progressive NMJ denervation (Niehues et al. 2015). Subsequent studies showed that mutant GlyRS sequesters tRNA^Gly^, causing ribosome stalling at glycine codons and triggering ISR activation (Mendonsa et al. 2021), a mechanism sufficient to drive NMJ loss in *Drosophila* (Mora et al. 2025). ISR activation has since been linked to NMJ denervation in multiple mutant *GARS1* models (*Gars*^*P278KY/*+^, *Gars*^*C201R/*+^ and *Gars*^*ΔETAQ/*+^), with increased motor neuron *Atf4* expression, eIF2α phosphorylation, and evidence that activity-dependent proteostatic stress exacerbates ISR-driven denervation via abnormal stress granule interactions (Cui et al. 2023; Spaulding et al. 2021). Genetic deletion of the ISR kinase *GCN2* rescues the *Gars*^*P278KY/*+^ phenotype, restoring NCVs, axon calibre and integrity, and soleus NMJ innervation – demonstrating that ISR activation is necessary rather than correlative for NMJ loss (Spaulding et al. 2021). These data identify translational stress in distal axons, which require continuous local synthesis of short-lived presynaptic and cytoskeletal proteins (Ravanidis et al. 2018), as a proximal driver of NMJ denervation.

A third convergent pathway involves axonal transport dysfunction. Mutant GlyRS disrupts microtubule-dependent transport through aberrant interactions with HDAC6; this leads to α-tubulin deacetylation and impaired trafficking of mitochondria and synaptic cargo, with HDAC6 inhibition improving NMJ innervation (Benoy et al. 2018; Mo et al. 2018). Indeed, a reduced density of mitochondria at *Gars*^*P278KY/*+^ presynaptic motor terminals perhaps reflects a failure of anterograde delivery (Spaulding et al. 2016). Aberrant associations with the extracellular domain of TrkB also impair BDNF-TrkB signalling endosome transport, a defect reversible upon supplementation of muscles with BDNF (Sleigh et al. 2023), which would otherwise dampen pro-survival transcriptional events from the soma (Scott-Solomon & Kuruvilla 2018). In addition, enhancement of circulating neurotrophin-3 (NT-3) via augmentation in muscle alleviates NMJ impairment in *Gars*^*P278KY/*+^ mice (Ozes et al. 2024). Together, these findings position NMJs as selectively vulnerable to chronic deficits in trophic support and bioenergetic capacity (Anagnostou & Hepple 2020; Dupuis et al. 2009).

In concert, experimental evidence identifies the NMJ as a selective, sensitive and mechanistically informative readout of mutant *GARS1* toxicity. Structural, physiological, developmental, and stress-related studies converge to show that NMJ dysfunction precedes axon loss and integrates multiple pathogenic pathways, while remaining partially reversible.

#### YARS1

Pathogenic variants in *YARS1*, which encodes tyrosyl-tRNA synthetase (TyrRS), cause dominant intermediate CMT subtype C (DI-CMTC), presenting with intermediate NCVs and progressive distal muscle weakness typically from adolescence (Jordanova et al. 2006). *YARS1* neuropathy closely parallels CMT2D in multiple core pathogenic principles – neomorphic residues confer a toxic gain-of-function on mutant TyrRS without mislocalisation in vivo, resulting in impaired axonal translation and transport, and aberrant binding to neuronal receptor protein TrkB, all independently from loss of aminoacylation activity (Bervoets et al. 2019; Blocquel et al. 2017; Hines et al. 2022; Niehues et al. 2015; Rhymes et al. 2024; Storkebaum et al. 2009). *Yars*^*E196K/E196K*^ mice, which possess the patient-sourced E196K mutation in homozygosity, also exhibit chronic activation of the ISR, as shown by elevated phospho-eIF2α activity – without replicating the stark neuropathology observed in CMT2D mice (Rhymes et al. 2024; Spaulding et al. 2021). Indeed, there is contrastingly no NMJ denervation or poly-innervation in *Yars*^*E196K/*+^ and *Yars*^*E196K/E196K*^ mutants at either 3 or 9 months in several different muscles across the body, nor any reduction in maximal grip strength. Additionally, myelination of both motor and sensory nerves also appears to be normal.

Nevertheless, homozygous *Yars* mutants display a motor-selective reduction in axon diameter, smaller lumbar motor neurons, a progressive decline in NCV and signalling endosome axonal transport, and age-related decline in muscle endurance (Hines et al. 2022; Rhymes et al. 2024). *Drosophila* larvae pan-neuronally expressing *yars*^*E196K*^ also displayed reduced NMJ length and bouton number, which were rescued by exclusion of the mutant protein from the nucleus, confirming additionally the mechanistic importance of transcriptional dysregulation caused by misinteractions between mutant TyrRS and nuclear proteins (Bervoets et al. 2019). Furthermore, the E196K mutation also disrupts a non-canonical actin-bundling function of TyrRS, producing presynaptic architectural defects and impaired synaptic vesicle organisation to directly implicate structural cytoskeletal instability in *Drosophila* NMJ maturation failure (Ermanoska et al. 2023; Morant et al. 2021).

Together, these studies indicate that NMJ dysfunction is present in *Drosophila yars*^*E196K*^ models, while mice with the same pathogenic variants appear less impacted, and do not show the same clear NMJ dysfunction conferred by mutant GlyRS in CMT2D mouse models – at least by 9 months of age. Still, as muscle-specific augmentation of BDNF rescued axonal endosome transport in *Yars*^*E196K/E196K*^ mice, evidence for non-cell autonomous destabilisation of the distal terminal as a contributor to neuropathy is indicated, while potentially signifying that aberrant interactions between different aaRS mutants and neuronal receptor proteins have multifarious context-dependent effects (Rhymes et al. 2024).

#### NEFL

*NEFL* encodes neurofilament light (NFL) chain, a key regulator of axonal cytoskeletal architecture, microtubule dynamics and organelle distribution (Kotaich et al. 2023). Pathogenic variants account for ~ 1% of CMT, causing dominantly inherited CMT2E, CMT1F or DI-CMT, with rarer recessive forms also reported (Abe et al. 2009; Stone et al. 2021; Yum et al. 2009). Across mutations and subtypes spanning all NFL domains, patient biopsies consistently indicate disrupted neurofilament organisation or axonal depletion as a shared pathogenic feature (Perez-Olle et al. 2004; Stone et al. 2021). A subset of patients show abundant neurofilament accumulations in sural nerve axons (Benedetti et al. 2010; Fabrizi et al. 2004; Stone et al. 2021), which resemble inclusions observed in ALS (Didonna & Opal 2019; Munoz et al. 1988); however, unlike in ALS, plasma/serum NFL cannot be used as a reliable biomarker in CMT (Campanari et al. 2019; Gaiani et al. 2017; Poesen et al. 2017; Rossor et al. 2022). These inclusion phenotypes contextualise divergent findings across NFL mouse models, which variably show prominent aggregation (Adebola et al. 2015; Shen et al. 2011) or minimal aggregation despite clear neuropathy (Dequen et al. 2010).

The NMJ has not been directly assessed in aggregate-forming *NEFL*^*N98S*^ models (Adebola et al. 2015; Lancaster et al. 2018; Rice et al. 2025). In contrast, *hNEFL*^*E397K*^ mice retaining endogenous mouse NFL show early accumulation of phosphorylated neurofilaments without evidence for hindlimb denervation, instead developing early proprioceptive sensory pathology with muscle spindle degeneration preceding classical CMT features (Shen et al. 2011; Villalón et al. 2017). Conversely, an endogenous *NEFL*^*E397K*^ knock-in model demonstrates early, progressive NMJ pathology. Multiple fore and hind limb muscles show significant denervation by 12 weeks, worsening by 12 months, with the greatest vulnerability observed in the tibialis anterior. NMJ loss precedes motor neuron degeneration and coincides with early functional electrophysiological abnormalities, including prolonged distal latency and reduced CMAP amplitude from P21. Subsequent CMAP stabilisation was attributed to compensatory NMJ sprouting, though direct evidence was not provided (Pérez-López et al. 2025).

Critically, aggregation-independent mechanisms are supported. *NEFL*^*N98S*^ iPSC-derived motor neurons exhibit impaired mitochondrial trafficking, functional hyperexcitability and reduced synaptic vesicle release without filament aggregation, linking axonal transport defects to distal synaptic failure (M. A. Saporta et al. 2015). Similarly, doxycycline-inducible *NEFL*^*P22S*^ mice show compromised mitochondrial transport and distal NMJ denervation without axon loss, while adult suppression of mutant NFL restores neurofilament distribution, rescues gastrocnemius innervation and improves motor function (Dequen et al. 2010).

This establishes a causal but reversible framework between mutant NFL disrupting cytoskeletal dynamics and axonal transport in driving distal mitochondrial depletion and denervation at axonal terminals, though model heterogeneity and complexity cautions against singular interpretations of the pathogenic impact of NFL.

#### DYNC1H1 and kinesins

Functionally consistent with NFL, pathogenic variants in other components of the axonal transport machinery, including dynein and kinesin motors, further implicate disrupted long-range transport as a recurrent contributor to axonal pathology in CMT. *DYNC1H1* encodes the heavy chain of cytoplasmic dynein, the motor protein driving retrograde axonal transport, and heterozygous missense mutations cause dominant CMT2O (Becker et al. 2020; Weedon et al. 2011). A heterozygous *DYNC1H1*^*H304R/*+^ knock-in mouse exhibits progressive motor deficits and nuanced NMJ alterations. Longitudinal analysis of gastrocnemius muscle reveals a biphasic NMJ phenotype, with structurally underdeveloped junctions at 1 month, apparent normalisation by 3 months, followed by age-dependent loss of complexity, including wider junction diameter, with reduced branch number and partial denervation between 6 and 12 months (Sabblah et al. 2018).

Gene-dosage effects further strengthen this link: homozygous *DYNC1H1*^*H304R/H304R*^ mice display earlier and more severe NMJ disruption in a wide range of morphological and innervation parameters in the same muscle at equivalent time points up to 12 months, alongside motor and sensory deficits (Nandini et al. 2019). Collectively, dynein mutant models provide strong evidence that impaired *DYNC1H1* disrupts NMJ development and long-term maintenance in CMT2O via a progressive degenerative mechanism without overt motor neuron loss.

Mutations in kinesin motors, including *KIF1A* and *KIF5A*, cause CMT and other diverse neuropathies (Zhao et al. 2001; Yonekawa et al. 1998; Cozzi et al. 2025). *Drosophila KIF1A* models demonstrate impaired NMJ formation and maturation, such as reduced excitation-–contraction coupling (Zhang et al. 2016; Bansal et al. 2025), whereas mammalian loss-of-function models are often too severe to reflect CMT, limiting synapse analysis (Yonekawa et al. 1998). *KIF5A*-associated NMJ pathology, such as distal motor unit degeneration, has been characterised primarily in ALS rather than CMT contexts, overall leaving understanding of the impact of dysfunction to these motor proteins on NMJ integrity suggested but comparatively sparse at present (Campbell et al. 2014; Rich et al. 2023; Soustelle et al. 2023).

#### Mitochondrial genes

Mitochondrial genes implicated in axonal CMT, including *MFN2* and *GDAP1*, support a model in which disrupted mitochondrial dynamics, transport, and/or bioenergetics predispose distal axonal terminals to degeneration and position the NMJ as a plausible site of selective vulnerability.

*Mitofusin-2* (*MFN2*) encodes a mitochondrial outer membrane GTPase required for mitochondrial fusion and axonal mitochondrial transport. Pathogenic *MFN2* variants are the most common cause of dominant axonal CMT, producing CMT2A through both loss- and gain-of-function mechanisms (Bombelli et al. 2014; Feely et al. 2011; Rizzuti et al. 2025; Züchner et al. 2004). Across cellular and animal models, *MFN2* dysfunction consistently disrupts mitochondrial transport while causing cytoskeletal dysfunction and distal axonal degeneration (Baloh et al. 2007; El Fissi et al. 2018; Misko et al. 2012; Picci et al. 2020).

Mouse studies, however, provide only weak evidence of direct NMJ dysfunction. Pharmacological mitofusin activation in HB9-*MFN2*^*T105M*^ mice is proposed to restore axonal mitochondrial transport and improve motor performance; however, claims of NMJ reinnervation and reversal of axonal die-back appear to exceed the supporting data (Franco et al. 2020, 2022). Other mouse models show either minimal structural denervation, or altered mitochondrial localisation at NMJs without comprehensive synaptic or functional assessment (Hines et al. 2023; Y. Zhou et al. 2019).

Stronger evidence derives from non-murine systems. Similarly to *NEFL* mutants, iPSC-derived *MFN2* mutant motor neurons exhibit hyperexcitability alongside mitochondrial transport defects, pointing to compromised synaptic transmission (M. A. Saporta et al. 2015). In *MFN2* mutant zebrafish, pre and postsynaptic NMJ abnormalities coincide with the onset of motor impairment (Chapman et al. 2013). Most compellingly, *MFN2*^*H361Y/*+^ rats exhibit progressive NMJ pathology in distal muscles, including presynaptic thinning, endplate shrinkage and denervation, with signs of attempted reinnervation, emerging after 6 months and worsening by 12 months. Upstream inhibition of *SARM1* prevents axonal and mitochondrial degeneration and restores NMJ innervation, demonstrating mechanistic linkage and therapeutic reversibility (Sato-Yamada et al. 2022).

*GDAP1* encodes a mitochondrial outer-membrane protein implicated in redox homeostasis (Pedrola et al. 2005) and is causative of clinically diverse axonal, demyelinating, dominant and recessive CMT phenotypes (e.g. CMT4A and CMT2K) (Baránková et al. 2007; Niemann et al. 2014). Two independently generated *GDAP1*^⁻/⁻^ mice, both lacking detectable protein, report opposing outcomes. One shows early motor deficits, axonal degeneration, motor neuron loss and age-dependent NMJ denervation alongside mitochondrial and calcium abnormalities (Barneo-Muñoz et al. 2015; Civera-Tregón et al. 2021; Fernandez-Lizarbe et al. 2019), while the other exhibits a milder, late-onset neuropathy with no early functional electrophysiological deficits, and no reported axon loss or NMJ pathology (Niemann et al. 2014), and the cause of this disparity remains unknown.

Other mitochondrial genes linked to neuropathy, sometimes identified/misidentified as axonal CMT2, including *DHTKD1*, *SLC25A46* and *PDK3* (CMTX6), demonstrate further associations between disrupted mitochondrial homeostasis and NMJ abnormalities. *DHTKD1* knockout (KO) or mutant mice develop clinical CMT-like phenotypes and progressive axonal degeneration with significant NMJ denervation (Luan et al. 2020; Xu et al. 2018). Knockdown (KD) or loss of *SLC25A46* is linked with disrupted mitochondrial dynamics as well as compromised synaptic structural maturation and connectivity via heightened poly-innervation at NMJs in *Drosophila* and murine models (Ali et al. 2020; Suda et al. 2018; Terzenidou et al. 2017). *PDK3 Caenorhabditis elegans* mutants produce presynaptic transmission defects consistent with mitochondrial-driven axonal and NMJ dysfunction (Kennerson et al. 2013; Narayanan et al. 2022; Perez-Siles et al. 2020). However, these cases do not clarify how synaptic mitochondrial dysfunction may mechanistically contribute to CMT progression; moreover, other mitochondrial genes more robustly associated with CMT, such as *COX6A1* and *AIFM1,* remain particularly under-investigated at the NMJ.

Together, mitochondrial CMT models converge on distal vulnerability as a pathogenic theme, but highlight that limited NMJ-focused analyses, model-specific discrepancies and inconsistencies currently constrain robust conclusions regarding synaptic dysfunction in this context.

#### IGHMBP2

Mutations in *IGHMBP2* cause both autosomal recessive axonal CMT2S and the more severe spinal muscular atrophy with respiratory distress type 1 (SMARD1). In contrast to CMT, SMARD1 presents in early infancy with rapid α motor neuron loss, profound denervation and fatal respiratory failure (Saladini et al. 2020; Villalón et al. 2018). *IGHMBP2* encodes an ATP-dependent RNA and DNA helicase involved in RNA metabolism, but how disruption of this function yields selective peripheral vulnerability and divergent disease severities remains unknown.

NMJ involvement is evident across *IGHMBP2*-associated neuropathies, though its pathogenic relevance differs markedly between SMARD1 and CMT2S. In SMARD1, NMJ pathology can accompany, follow or remain absent alongside severe motor neuron degeneration, whereas in CMT2S, NMJ destabilisation emerges as a consistent and early feature, supporting a distal mechanism of synaptic dysfunction.

In SMARD1, interpretation of NMJ pathology is complicated by model heterogeneity. Classical mouse models, including nmd, R604X and D564N, develop early motor neuron loss and respiratory failure (Grohmann et al. 2004; Smith et al. 2022; Villalón et al. 2018). Human biopsies show widespread NMJ denervation concurrent with axonal loss and failed regeneration (Diers et al. 2005). However, some models, notably nmd, display relatively preserved NMJ morphology and transmission despite motor neuron degeneration, with terminal Schwann cell sprouting suggestive of compensatory remodelling rather than primary synaptic failure (Grohmann et al. 2004; Krieger et al. 2013). More recent SMARD1 models show preserved diaphragmatic NMJ structure despite respiratory failure, alongside pronounced distal axonal degeneration, NMJ denervation, muscle atrophy and electrophysiological abnormalities – indicating selective vulnerability across muscle groups rather than uniform NMJ collapse (S. Holbrook 2024; Torres et al. 2025; Villalón et al. 2018).

By contrast, *IGHMBP2* models that better phenocopy CMT2S, including ΔE365, H922Y and Y918C, show juvenile- or adult-onset axonal neuropathy, minimal motor neuron loss and no early respiratory involvement (Martin et al. 2022; Ricardez Hernandez et al. 2025). Across ΔE365 and Y918C models, progressive NMJ denervation emerges in distal muscles and correlates with weakness and atrophy. Importantly, distal NMJ loss exceeds proximal motor axon loss, strongly supporting primary synaptic vulnerability rather than neuron loss-driven denervation (Martin et al. 2022). Human iPSC-derived motor neuron-muscle microfluidic systems carrying CMT2S-associated *IGHMBP2* variants additionally demonstrate early synaptic functional hyperexcitability and later transmission failure, with functional rescue following antisense oligonucleotide treatment (Smieszek et al. 2025).

Mechanistically, partial or aberrant *IGHMBP2* activity in CMT2S may induce chronic RNA-processing or translational stress (Vadla et al. 2024). SMARD1 mutations predominantly affect the helicase domain and are typically missense, whereas CMT2S mutations often involve truncating variants and result in greater residual protein expression (Saladini et al. 2020). This distinction may underlie selective severity and peripheral vulnerability between the conditions, potentially paralleling other RNA-processing CMT genes with non-cell autonomous effects on the NMJ, such as *GARS1* in CMT2D (Grice et al. 2015; Mendonsa et al. 2021; Niehues et al. 2015; Spaulding et al. 2021).

Collectively, these findings indicate that *IGHMBP2* mutations produce qualitatively distinct patterns of NMJ involvement in CMT2S and SMARD1, with mutation-specific variants beginning to help explain divergent pathogenic mechanisms and the selective vulnerability of the NMJ in CMT2S.

#### Axonal CMT: translation, transport, trophic support and transmission

Across axonal CMT2 subtypes, the NMJ emerges as a recurring site of selective, distal vulnerability rather than a uniform or non-specific consequence of general neuronal dysfunction (Fig. 2C, Supplementary Table 1). In a subset of models, NMJ abnormalities are length-dependent, progressive, and precede distal axonal degeneration (notably *GARS1* and *IGHMBP2*), consistent with the exceptional metabolic and signalling demands imposed on long motor neurons and supportive of their role as early sites of pathology. Importantly, in *IGHMBP2*-associated disease, this pattern is specific to CMT2S-linked variants, underscoring CMT-specific mechanisms of NMJ dysfunction. As well as degenerative alterations, additional deficits in NMJ maturation in CMT are also indicated via *GARS1* and *DYNC1H1* models. Among axonal CMT genes, *GARS1* provides the most comprehensive template, linking robust in vivo structural, functional, and longitudinal NMJ analyses to defined molecular mechanisms, while many other genes remain, so far, less completely evaluated.

The identified NMJ dysfunction in axonal CMT2 suggests convergence along four intersecting mechanistic axes. First, chronic RNA-processing or translational stress (e.g. *GARS1*, *IGHMBP2*, *YARS1*) may impair local protein homeostasis required for synaptic maintenance. Second, defects in axonal transport and cytoskeletal integrity (e.g. *GARS1*, *YARS1*, *HSPB1*, *NEFL*, *DYNC1H1*, KIFs) limit cargo delivery to distal terminals and destabilise axon structural support. Third, mitochondrial and trophic insufficiency is conceptually implicated (e.g. *MFN2*, *GDAP1*, *GARS1*) to compromise energy supply, pro-survival signalling and the capacity for adaptive plasticity at distal terminals. Fourth, direct neurotransmission defects, most clearly demonstrated in *GARS1* mice in vivo and partially supported by in vitro studies of *IGHMPB2*, *NEFL* and *MFN2*, illustrate firing dysfunction as a possible contributor to downstream motor deficits. Reinnervation or regenerative indications are also not currently reported across CMT2 once NMJs are compromised, suggesting that inadequate regenerative capacity contributes to sustained motor deficits.

However, the strength of evidence varies markedly between genes, with limited longitudinal NMJ analyses, inconsistent electrophysiological readouts to deconvolve structural from functional deficits, and heavy reliance on single models, constraining causal inference. Critically, beyond *GARS1*, NMJ investigation in axonal CMT is rarely temporally coupled to axonal integrity measurements across a range of models or to extensive molecular elucidation, limiting resolution of understanding for the depth, significance and progression of synaptic dysfunction in relation to axonal compromise. Together, these patterns position the NMJ as an unevenly explored, but mechanistically informative and often early pathological target in axonal CMT, motivating expanded, integrative studies to clarify its contribution to distal axonal pathology.

### Demyelinating CMT

Demyelinating CMT arises from primary Schwann cell and myelin dysfunction, leading to slowed nerve conduction and secondary axonal impairment (Fridman & Saporta 2021). CMT1 subtypes are typically autosomal dominant and most commonly involve alterations in major myelin genes, particularly *PMP22* and *MPZ*, which encode proteins essential for myelin compaction, stability, and Schwann cell-axon interactions (Niemann et al. 2006; Rossor et al. 2013). In contrast, CMT4 subtypes are largely autosomal recessive, affecting genes that regulate Schwann cell differentiation, membrane trafficking, lipid metabolism and myelin maintenance (e.g. *SH3TC2*, *MTMR2/13*, *FIG4*) (Berger et al. 2006; A. S. D. Saporta et al. 2011). These disorders encompass multiple strands of vulnerability, including primary hypomyelination and nodal disruption, as well as Schwann cell proteostatic stress. Additionally, Schwann cell dysfunction can engender secondary axonopathy, rendering axons vulnerable via disruption to myelination, metabolic support and signalling required for axonal integrity. Dysmyelinated axons show early cytoskeletal disruption, swelling, altered gene expression, and impaired axonal transport that precede overt axon loss (de Waegh et al. 1992; Griffiths et al. 1998; Nave et al. 2007; Yin et al. 2004). Loss of myelin-dependent nodal organisation further increases energetic demand and disrupts ion homeostasis to amplify axonal stress (Gerevich et al. 2023; Hamada & Kole 2015; Moss et al. 2021).

In concert, these perisynaptic changes due to Schwann cell dysfunction can propagate structural denervation and thus plausibly contribute to progressive transmission failure at the NMJ (Moss & Saxena 2025). Additionally, there is evidence chronic Schwann cell stress can destabilise NMJs without requiring prior axonal disintegration (Arthur-Farraj & Coleman 2021; Yin et al. 2004), relating to indications of functional denervation. As a form of functional denervation, conduction block is more commonly described in acquired rather than genetic demyelinating neuropathies (Kaji 2003; Nagappa et al. 2025), but there is substantial clinical evidence supporting its occurrence in demyelinating CMT (Echaniz-Laguna 2015; Hu et al. 2018; Kaku et al. 1993; Krajewski et al. 2000; J. Li et al. 2002; J. Li & Stefanelli 2020; Manganelli et al. 2016; Murphy et al. 2011; Nagappa et al. 2025; Villar-Quiles et al. 2021), as well as in demyelinating CMT mouse models (Baloh et al. 2009; Court et al. 2008a; Deres et al. 2021; Saifetiarova et al. 2017; Wrabetz et al. 2006).

Collectively, despite clear relations of axonal pathology to Schwann cell dysfunction in demyelinating CMT and independent indications of Schwann cell-driven NMJ dysfunction, NMJs appear a less direct target in demyelinating CMT and have so far been less systematically interrogated than in axonal CMT. As synapses are now recognised as shared sites of vulnerability across numerous neurodegenerative disorders, extending this framework to peripheral myelin diseases broadens the spectrum of conditions in which synaptic protection at the NMJ may offer therapeutic benefit (Ang et al. 2010; Henstridge et al. 2016; Wishart et al. 2006).

#### PMP22

*Peripheral myelin protein-22* (*PMP22*) is expressed by myelinating Schwann cells and enriched in compact peripheral myelin, where it contributes to myelin stability and internodal membrane organisation (Boutary et al. 2021; J. Li et al. 2013). Alterations in *PMP22* cause a spectrum of peripheral neuropathies accounting for over 50% of CMT cases, including dominant CMT1A due to *PMP22* duplication, and the much rarer CMT1E caused by point mutations (Cesaroni et al. 2025; J. Li et al. 2013). Across this allelic spectrum, *PMP22* pathology is unified by chronic Schwann cell dysfunction that predisposes neurons to distal axonal and neuromuscular failure.

In CMT1A, *PMP22* overexpression disrupts proteostasis, causing endoplasmic reticulum (ER) stress, Schwann cell dysfunction and dysmyelination with secondary axonal degeneration (Krajewski et al. 2000; Stavrou et al. 2023). Patients exhibit early axonal involvement correlating with clinical severity (Krajewski et al. 2000; Robertson et al. 2002), while rodent models show a gene-dosage relationship: high-copy C22-PMP22 overexpression mice develop severe demyelination with later axon loss, whereas low-copy C3-PMP22 mice exhibit milder myelin defects and delayed secondary axon degeneration (Huxley et al. 1996; Robertson et al. 2002; Sereda et al. 1996). Recently, CMT1A models have expanded to 3D iPSC-derived myelinating organoids and humanised mice, with discoveries of species-specific alternative *PMP22* splicing (Taruta et al. 2025; Van Lent et al. 2023; Visigalli et al. 2016), highlighting the complexity of *PMP22* pathology and the need for careful model selection and interpretation.

Despite heterogeneity in myelin and axonal pathology, unique NMJ dysfunction is a consistent downstream feature in *PMP22* overexpression models. C22-PMP22 mice exhibit early terminal sprouting in soleus and extensor digitorum longus (EDL) muscles that inversely correlates with loss of occupied NMJs, progressing to muscle atrophy and near 50% loss of hindlimb muscle NMJs by 6-8 months. These patterns are indicative of ineffective denervation-reinnervation cycles that fail to stabilise synapses despite preserved regenerative capacity (Ang et al. 2010). Importantly, NMJ instability in C22-PMP22 mice arises slowly in the context of severe dysmyelination, with minimal axon loss and no classic denervation hallmarks like extrajunctional AChR deposition by P21, a time when hindlimb weakness is already present – supporting functional denervation driven by Schwann cell pathology over primary axon degeneration (Deres et al. 2021).

Mechanistically, NMJ destabilisation in CMT1A reflects chronically disrupted Schwann cell signalling required for axonal maintenance. In CMT1A rats, early NRG1 type III-ERBB2/3 impairment together with sustained MEK-ERK activation precedes gastrocnemius NMJ loss by six months (Fledrich et al. 2014) – potentially indicating NRG1/ErbB signalling is essential for maintaining normal AChR numbers at the NMJ (Schmidt et al. 2011). Soluble NRG1 supplementation or PXT3003 polytherapy modestly improves NMJ innervation (potentially due to improved Schwann cell signalling and axon metabolic support); however, restricting axonal degeneration via *Wlds* transgene, nicotinamide, or *SARM1* deletion in the CMT1A rat or C3-PMP22 mouse does not fully restore motor function when myelin integrity remains compromised, suggesting that NMJ loss in CMT1A is largely a metabolic or trophic, Schwann cell-dependent process rather than acute axon self-destruction (Meyer Zu Horste et al. 2011; Moss et al. 2022).

Interestingly, the novel, recent application of electrical impedance myography (EIM) in C3-PMP22 mice provides a non-invasive, sensitive readout of progressive NMJ instability and muscle atrophy signatures that precede overt behavioural weakness, supporting translational relevance of EIM in CMT as a non-invasive tool for monitoring disease progression (Sanchez & Rutkove 2017; Taruta et al. 2025).

In CMT1E caused by *PMP22* point mutations, NMJ pathology shows both overlap and divergence from CMT1A duplication models. Well established Trembler and Trembler-J (Tr-J) mice carry missense mutations (Gly160Asp and Leu16Pro) that promote *PMP22* aggregation, ER retention, UPR activation and severe Schwann cell proteotoxic stress along with additional loss of native *PMP22* (Bosco et al. 2021; D’Urso et al. 1998; Okamoto et al. 2013; Tobler et al. 2002).

In heterozygous Tr-J mice, both foundational and newer longitudinal work establish that distal demyelination is accompanied by structural terminal axonal thinning, robust GAP43-positive axonal sprouting and progressive, length-dependent NMJ denervation that preferentially affects slow-twitch muscles, and closely parallels motor decline (Gale et al. 1982; J. R. Nicks et al. 2013). Single-fibre EMG reveals increased jitter and transmission block preceding overt denervation, with transient improvement following acetylcholinesterase inhibition, indicating early functional synaptic transmission instability (Meekins et al. 2007). Therapeutic dissociation is evident, as rapamycin-mediated autophagy enhancement improves myelin integrity and reduces *PMP22* aggregation, but fails to rescue muscle atrophy, motor performance or NMJ innervation (J. Nicks et al. 2014).

More severe homozygous Tr-J mice additionally illustrate that, despite complete congenital hypomyelination and early lethality, NMJs remain anatomically intact across multiple muscles, with preserved synaptic markers and no overt denervation (Scurry et al. 2016). Instead, NMJs display delayed maturation via reduced endplate volume, diminished junctional folding, and immature plaque-like morphology, alongside profound presynaptic failure marked by reduced quantal content, decreased mEPP frequency, and activity-dependent transmission decline (Scurry et al. 2016).

Jointly, these findings robustly demonstrate that chronic Schwann cell and myelin dysfunction via *PMP22* disturbance is sufficient to destabilise NMJs through impaired conduction, aberrant sprouting, and ineffective regenerative support, even in the absence of overt axonal structural collapse. Moreover, missense variants further strengthen evidence that maturation and primary synaptic transmission failure can precede axon degeneration, revealing a distinct mode of NMJ vulnerability under conditions of extreme Schwann cell dysfunction that potentially echoes temporal patterns and mechanisms executed by toxic gain-of-function proteins, as established in CMT2D.

#### MPZ

*MPZ* encodes P0, a Schwann cell-specific glycoprotein and the dominant structural component of peripheral myelin. Mutations in *MPZ* cause one of the most common and phenotypically diverse forms of CMT, including dominant CMT1B and more recently axonal CMT2J, along with Congenital Hypomyelinating Neuropathy (CHN) and Dejerine-Sottas syndrome (DSS) (McCulloch et al. 2024; Scherer & Wrabetz 2008; Stavrou et al. 2021a).

Across the broad spectrum of *MPZ* mutant models that segregate into several distinct pathogenic classes with variable impacts on myelination and protein processing, NMJs are rather variably affected.

The clearest evidence linking *MPZ* dysfunction to strong NMJ destabilisation derives from *MPZ* overexpression models that induce severe hypomyelination (Wrabetz et al. 2000). Longitudinal assessment demonstrates that NMJs initially form normally but undergo considerable progressive denervation, with normal innervation dropping in the gluteus maximus from 75% at P15, to 3% at P90 without loss of motor neurons. AChR fragmentation and terminal Schwann cell sprouting reveal additional morphological alterations, with indications also of presynaptic functional transmission defects, including reduced vesicle recycling and quantal content. Importantly, these synaptic changes precede axonal degeneration, and restoration of myelination rescued NMJ innervation and transmission – implicating sustained dysmyelination of preterminal axons as the primary driver of synaptic failure (Yin et al. 2004). Interestingly however, denervation of NMJs did not conform to a general length-dependent axonopathy as the longest nerves were not affected first.

Contrastingly, endogenous gain-of-function *MPZ* mutations that exhibit ER retention of mutant P0, chronic UPR activation and dysregulated Schwann cell transcription (Arthur-Farraj & Coleman 2021; Pennuto et al. 2008; M. A. C. Saporta et al. 2012; Wrabetz et al. 2006), produce subtler NMJ phenotypes in line with milder dysmyelination. In *MPZ*^*R98C*^ knock-in (KI) mice, NMJs in both distal soleus and gastrocnemius, as well as proximal triangularis sterni, muscles subsequently show reduced myelin coverage of preterminal axons by 3 months, without overt denervation. Partial phenotypic rescue following curcumin-mediated UPR attenuation supports a causal role for Schwann cell stress pathways, though interpretation is limited by curcumin pleiotropy (Patzkó et al. 2012).

The *MPZ*^*T124M*^ mutation causing axonal CMT2J further decouples NMJ dysfunction from classical demyelination or axonal loss. These mice show minimal NCV slowing or myelin disruption, but reduced CMAP amplitudes, disturbed paranodal organisation, and substantial, progressive loss of large-calibre axons, as well as demyelinating fibres in the sciatic nerve that show signs of degenerative axonal swelling or “regenerative clusters” (Shackleford et al. 2022). Defective retrograde axonal transport of endosomes and swollen axonal mitochondria are also observed, potentially as a consequence of impaired Schwann cell trophic support at nodal domains (Claessens 2025). Despite this, distal hind paw muscle NMJs intriguingly remain structurally innervated at 12 months, while *SARM1* deletion restored axonal degeneration, but not CMAP amplitudes – pointing toward a dissociable relationship between structural NMJ preservation and motor function in relation to axon loss (Claessens 2025).

Taken together, *MPZ*-associated neuropathies reveal that Schwann cell pathology can generate multiple, mechanistically distinct NMJ states, ranging from early synaptic destabilisation with severe dysmyelination, to preserved structural innervation despite axon loss. This challenges simple models that equate NMJ integrity with axonal survival, myelin status, or motor function.

#### EGR2

Early Growth Response 2 (EGR2) is a central transcription factor governing peripheral myelination, associated with a varied spectrum of neuropathies including both dominant and recessive CMT1D and CMT4E, DSS, and CHN (M. A. Saporta et al. 2023). While classically viewed as a disorder of Schwann cell transcriptional failure causing hypomyelination and severely reduced conduction velocities (Baloh et al. 2009; Nagarajan et al. 2001), recent cases also demonstrate axonal CMT phenotypes, positioning *EGR2* as a mixed demyelinating-axonal disease gene (Echaniz-Laguna et al. 2023; Sevilla et al. 2015).

Mouse models indicate that *EGR2* dysfunction precipitates NMJ phenotypes secondary to conduction insufficiency rather than primary axon loss. *EGR2*^*I268N*^ homozygous mice, which possess a CMT4E patient-derived point mutation, display phenotypes more reminiscent of CHN, in which Schwann cells fail to initiate postnatal myelination due to mutant proteins conformationally unable to activate transcription of target genes, causing profound conduction block and weakness. At points of near paralysis, *EGR2*^*I268N/I268N*^ mice display preserved axon counts and fully occupied NMJs with terminal sprouting in the EDL muscle, consistent with functional rather than structural denervation (Baloh et al. 2009). Similarly, Schwann cell-specific *EGR2* deletion induces chronic conduction block and progressive postsynaptic remodelling, including AChR dispersion in both the fast-twitch EDL and slow-twitch soleus muscles, without early axonal loss (Zotter 2020).

Disrupting upstream regulators (*TFEB/TFE3*) also impairs Schwann cell repair and prevents resolution of NMJ denervation post-injury, further reinforcing the dependence of NMJ maintenance on intact *EGR2*-dependent myelin programs (Patel 2023). Collectively, these studies indicate that NMJ dysfunction in *EGR2*-related neuropathies arises due to sustained myelination failure rather than axon degeneration or withdrawal.

#### SH3TC2

Mutations in *SH3TC2* cause CMT4C, which is the most prevalent form of recessive demyelinating CMT and is characterised by early hypomyelination, dramatically slowed NCVs and variable clinical severity, frequently with cranial nerve involvement and scoliosis (Fridman & Saporta 2021; Ozes et al. 2024). *SH3TC2* is selectively expressed in Schwann cells, where it regulates endosomal recycling and ERBB2 receptor internalisation – processes essential for myelin maintenance, nodal organisation and axonal support (Arnaud et al. 2009; Rehbein et al. 2020).

Evidence for NMJ pathology in CMT4C is present, but limited and heterogeneous. Preserved NMJ morphology and neuromuscular transmission after stimulation were reported in plantaris muscles of *SH3TC2*^−/−^ mice at 3.5 months, despite established demyelinating neuropathy, suggesting that Schwann cell dysfunction alone may be insufficient to structurally destabilise hindlimb NMJs at early stages in this model. However, preterminal conduction block was substantially observed, indicating NMJs in *SH3TC2*^−/−^ mice may retain the capacity to fire, but in fact experience early and length-dependent failures in action potential propagation and states of functional denervation (Morelli et al. 2017).

In contrast, significant NMJ structural alterations in gastrocnemius muscles of *SH3TC2* knockout mice were identified at subsequent time points of 8 months, including reduced synaptic contact area, postsynaptic fragmentation and abnormal presynaptic branching, without overt axonal loss. Some molecular changes were concomitantly observed, including upregulation of denervation-associated markers, such as the γ AChR subunit and nerve growth factor, as well as proteomic changes in ECM components implicated in NMJ stability; though most canonical denervation and reinnervation pathways remained unchanged and sample sizes were small (Cipriani et al. 2018). Greater denervation in more distal hindpaw lumbrical muscles at 6 months has also now been reported, potentially supporting length- and age-dependent vulnerability. Importantly, muscle-targeted NT-3 delivery partially restored NMJ innervation and improved motor performance, demonstrating therapeutic reversibility (Ozes et al. 2024).

Overall, *SH3TC2*-associated CMT4C shows emerging, but complex and inconsistently assessed, NMJ dysfunction that likely occurs secondary to myelination perturbation. This may point to progressive age- and distal length-dependent susceptibilities with contributions of both functional and structural denervation, but further multiplexed and multi-site longitudinal studies are required to confirm these varied pathological relationships.

#### PRX, FGD4 and CNTNAP1

Emergent but limited evidence for NMJ dysfunction is also present across additional demyelinating CMT genes predominantly governing myelin stability and organisation, including recessive *PRX* (CMT4F), *FGD4* (CMT4H) and *CNTNAP1*/*Caspr* (severe demyelinating CMT1-CHN spectrum) (Y. Chen et al. 2019; Freed et al. 2019; Kijima et al. 2004; Lesmana et al. 2019; Zis et al. 2017). Detailed yet isolated studies appear to suggest NMJ pathology can arise predominantly as a secondary consequence of dysmyelination, sustained myelin instability and conduction failure, rather than due to earlier hypomyelination or alternate axonal withdrawal.

In periaxin deficiency, myelination initiates normally but is progressively destabilised. In *PRX*^*⁻/⁻*^ mice, segmental demyelination of preterminal motor axons induces pronounced triangularis sterni NMJ remodelling. These changes include excessive preterminal branching structurally, axon thinning and focal swellings arising directly from demyelinated internodes proximal to the endplate, without tSC activation, poly-innervation, denervation or axon loss (Court et al. 2008a). Electrophysiology demonstrates preserved low-frequency transmission, but frequency-dependent conduction block at branch points, establishing that local preterminal demyelination alone is sufficient to impair NMJ function post-development as a pattern closely mirroring early-stage *SH3TC2* pathology.

*FGD4* loss similarly produces progressive Schwann cell dysfunction rather than early myelination failure. Schwann cell-specific *FGD4* knockout mice exhibit early distal myelin abnormalities linked to disruption of the NRG1 type III–ERBB2/3 signalling axis, followed by late-onset axonal degeneration and increases in partially and fully denervated gastrocnemius NMJs by 12 months (El-Bazzal et al. 2023). Other demyelinating CMT genes like *NDRG1* (CMT4D) also converge on Schwann cell NRG1-1-ERBB2/3 signalling, where models recapitulating peripheral neuropathy are noted in mice and dogs, but NMJs have not yet been examined (Echaniz-Laguna et al. 2007; Jiang et al. 2022; King et al. 2011; Okuda et al. 2004; Skedsmo et al. 2021).

In *CNTNAP1*/*Caspr* knockout models, NMJ development is initially intact, but progressive paranodal and juxtaparanodal disorganisation causes Kv1 channel mislocalisation, conduction slowing and distal muscle atrophy. By adulthood, tibialis anterior NMJs show reduced postsynaptic AChR area, partial and complete structural denervation, abnormal preterminal branching, presynaptic vesicle defects and early mitochondrial abnormalities via ultrastructural analysis preceding overt synaptic failure (Saifetiarova et al. 2017). Postnatal neuronal *CNTNAP1* re-expression restores paranodal architecture and motor function, demonstrating reversibility of axonal domain defects (Chang et al. 2023).

Though temporal and regional patterns of NMJ vulnerability remain unclear, collectively these genes support a model in which myelination is initially established, but progressive Schwann cell or axonal domain instability may contribute to driving NMJ dysfunction.

#### GJB1

*GJB1* encodes connexin 32 (Cx32), a gap junction protein expressed by myelinating Schwann cells within non-compact myelin that supports axonal metabolic and ionic exchange (Fridman & Saporta 2021; Scherer et al. 1998). Mutations cause X-linked intermediate CMT1X, the second most common form of CMT after CMT1A, with males typically more severely affected (Stavrou et al. 2021a, ). Broadly comparable disease severity across null, nonsense, and missense mutations indicates demyelinating loss of gap junction function as the dominant pathogenic mechanism (Record et al. 2023; Shy et al. 2007). However, many missense variants mislocalise to the ER or Golgi and induce proteostatic stress, suggesting additional toxic gain-of-function components distinct from simple channel loss, more analogous to *MPZ* and *PMP22* pathology (Chu et al. 2022; D’Urso et al. 1998; Kleopa et al. 2012; Tobler et al. 2002; Wrabetz et al. 2006).

Cx32-null mice establish a canonical sequence of progressive demyelination followed by secondary axonal degeneration, particularly in motor fibres (Scherer et al. 1998). Distal NMJ denervation and muscle fibre atrophy are evident in hindlimb flexor digitorum brevis muscles by 12 months, coinciding with macrophage infiltration and motor deficits (Groh et al. 2010). Genetic or pharmacological suppression of macrophages preserves axons, reduces NMJ denervation, and improves motor performance, despite only partial or transient correction of demyelination (Groh et al. 2010; Klein et al. 2015). These findings indicate that inflammation-driven axonopathy, rather than myelin loss alone, is a determinant of distal NMJ failure, although evidence for compensatory terminal Schwann cell sprouting or reinnervation as proposed mechanisms for myelin-independent contributions remain here insufficiently demonstrated.

More recent work extends NMJ analysis to EDL muscles and missense variants, showing partial denervation in Cx32-null and R75W knock-in mice. Long-term treatment with the Hsp90 inhibitor cemdomespib slows NMJ withdrawal, improves myelin thickness, and preserves motor function (Kaur et al. 2023; Lang et al. 2025); but NMJ or CMAP rescue is incomplete, variably quantified, or assessed in limited muscles and single timepoints. Gene replacement strategies restore myelination and conduction even after disease onset but have not yet indicated NMJ restoration, and underscore the need to tailor therapies to distinct pathogenic mechanisms (Kagiava et al. 2019; Pisciotta & Pareyson 2023).

In concert, *GJB1* models more clearly support axonal pathology and NMJ destabilisation secondary to Schwann cell dysfunction, modulated by inflammation and proteostatic stress; but the absence of systematic, longitudinal NMJ analyses across diverse model classifications confounds direct mechanistic understanding as yet.

#### Demyelinating CMT: secondary NMJ instability, sprouting and regeneration

Across demyelinating CMT subtypes, NMJ dysfunction is emerging as a recurrent feature of secondary destabilisation following a failure of Schwann cell support (Fig. 2C, Supplementary Table 1). In contrast to axonal CMT, NMJ abnormalities frequently arise in the setting of preserved axonal continuity but impaired conduction and preterminal demyelination or metabolic insufficiency. This results in functional denervation before or without overt axon loss (e.g. *PMP22* duplication or missense mutation, *EGR2, SH3TC2, PRX*), indicating that NMJ dysfunction can arise primary to or independently from frank degeneration. However, direct electrophysiological assessment of synaptic transmission remains limited for many demyelinating subtypes, restricting evaluation of the full functional impact of these alterations.

Mechanistically, chronic Schwann cell stress represents a central driver of NMJ instability, including toxic gain-of-function effects of misfolded or overexpressed myelin proteins (e.g. *PMP22*, *MPZ*, *GJB1*), or indications of aberrant NRG1 type III–ERBB2/3 signalling (e.g. *PMP22*, *SH3TC2*, *FGD4*, *NDRG1*). Severe hypomyelination or myelination failure demonstrates early dysfunction even in the presence of apparently intact NMJs (e.g. *EGR2, SH3TC2*) not fully reminiscent of CMT phenotypes, while structural denervation, axonal disintegration and NMJ loss is observed in some models, especially at later stages, subsequent to dysmyelination (e.g. *MPZ* overexpression, *FGD4*, *SH3TC2*, *CNTNAP1*, *GJB1*).

Unlike axonal CMT, demyelinating models more frequently exhibit axonal sprouting and attempted reinnervation (e.g. *PMP22*, *EGR2*, *MPZ* overexpression), indicating at least partially retained regenerative capacity; however, these responses are not fully characterised, and appear insufficient to sustain stable synaptic function, implying repeated cycles of denervation and ineffective repair. Reliance on single experimental timepoints may therefore underestimate conclusions of NMJ instability. Therapeutically, distinctions underscore that strategies aimed solely at preventing axon degeneration are unlikely to preserve NMJ function in demyelinating CMT, necessitating future approaches that target chronic Schwann cell dysfunction, myelin instability and associated stress pathways to stabilise neuromuscular connectivity.

### Endolysosomal genes common to axonal and demyelinating CMTs

A substantial subset of CMT genes converge on endolysosomal and membrane trafficking pathways across both demyelinating and axonal subtypes, yet interrogation of mammalian NMJs in this context remains sparse. Mouse models of demyelinating variants typically highlight Schwann cell pathology and myelin abnormalities, as seen with myotubularin-related phosphatases (*MTMR2*, *MTMR13*, *MTMR5*) in CMT4B (Mammel et al. 2021; Previtali et al. 2007; Robinson et al. 2008) and *FIG4* in CMT4J (Lauerova et al. 2025; Vaccari et al. 2015). However, demyelinating subtypes also exhibit axonal dysfunction due for instance to paranodal abnormalities, hypothesised to disrupt axonal transport, as in *LITAF* mutant models (CMT1C) – again implicating Schwann cell-driven endosomal stress in downstream axonal impairment (Edgar et al. 2020; S. M. Lee et al. 2013). Consistent with this, *FIG4* and *LITAF* operate within a shared late endosomal pathway suggestive of common dysfunction, and *Drosophila FIG4* models show pronounced NMJ abnormalities, including reduced synaptic branching, bouton loss, axonal targeting defects, and impaired locomotion (Kyotani et al. 2016; Shimada et al. 2020).

Mutations in the ubiquitin ligase *TRIM2* conversely cause axonal CMT2R (Magri et al. 2020; Pehlivan et al. 2015; M. Xiao et al. 2022) where knockout mouse models display preserved myelination but axonal degeneration (J. J. Li et al. 2020). Among further E3 ubiquitin ligase CMT2 genes, *LRSAM1* loss-of-function mice fail to fully recapitulate human pathology, and NMJs display only subtle postsynaptic morphological alterations at 5, but not 12, months (Bogdanik et al. 2013; Hakonen et al. 2017; Nicolaou et al. 2013).

Collectively, these findings indicate convergence of endolysosomal and ubiquitin proteasome pathways across demyelinating and axonal CMT subtypes, where axonal or synaptic dysfunction is partially suggested mainly through non-mammalian systems, but remains poorly characterised at mammalian NMJs. Evidence from ALS and related neuromuscular diseases demonstrates that defects in endolysosomal vesicle turnover can directly drive NMJ pathology while even being linked specifically to “dying-back” neuropathy (Coyne et al. 2017; McLean et al. 2024; Yaron & Schuldiner 2016), and that the ubiquitin proteasome system is essential for maintaining skeletal muscle integrity (Bachiller et al. 2020) – together underscoring the need for targeted NMJ focused investigation of these pathways in CMT.

### CMT-causing genes in related neuropathies

The impact of mutations in several CMT-linked genes on the NMJ are better studied in other neuromuscular disease contexts, providing comparative insights into mechanisms of synaptic dysfunction potentially relevant to CMT.

#### SYT2

*SYT2* encodes synaptotagmin-2, the principal presynaptic calcium sensor mediating fast acetylcholine release at the NMJ (Q. Zhou et al. 2017). Dominant *SYT2* mutations cause a non-progressive distal motor syndrome often misdiagnosed as axonal CMT2 or dHMN, whereas recessive variants lead to severe presynaptic congenital myasthenic syndromes (CMS) (Fontana et al. 2025; Herrmann et al. 2014; Whittaker et al. 2015).

Pathogenic *SYT2* variants illustrate a disease spectrum where NMJ dysfunction is primary and axonal degeneration is minimal or absent. Electrophysiological assessments consistently show presynaptic transmission failure – marked decrement on repetitive nerve stimulation and abnormal jitter on single-fibre EMG – often without significant motor axon loss (Fionda et al. 2021; McMacken et al. 2023). *SYT2* knockout mice and patient biopsies show reduced *SYT2* expression at NMJs, compensatory *SYT1* upregulation, and disruption of pre and postsynaptic architecture to directly implicate presynaptic functional compromise via disrupted synaptic vesicle release machinery (Bauché et al. 2020; Pang et al. 2006).

*SYT2*-associated disease models therefore challenge axon-centric conceptualisations of CMT by demonstrating that profound distal weakness and “CMT-like” neuropathy phenotypes can arise from isolated NMJ transmission failure. While *SYT2* is not a canonical CMT gene, it underscores the NMJ as a critical site where defects in synaptic release can phenocopy CMT2, and proves that distal weakness alone cannot be assumed to reflect axonal degeneration (Herrmann et al. 2014; McMacken et al. 2023).

#### SLC12A6

*SLC12A6*, a dominant CMT2-linked gene encoding the K⁺-Cl⁻ co-transporter KCC3 (Ando et al. 2022), has not been studied at the NMJ in CMT models. However, neuron-specific *SLC12A6*⁻/⁻ mice modelling Andermann syndrome show early NMJ defects and presynaptic transmission abnormalities. Impaired neurotransmission precedes overt denervation and axonal degeneration, contributing causally to locomotor deficits. Motor neuron hyperexcitability additionally drives NMJ denervation, while experimentally reducing firing frequency slows synaptic loss and restores tibialis anterior muscle innervation (Bowerman et al. 2017). As it is not fully determined whether excitability is toxic, inconsequential, or protective of motor neuron health in different contexts (Gunes et al. 2020; Leroy & Zytnicki 2015; Odierna et al. 2024; Saxena et al. 2013), these findings strongly motivate targeted studies to test whether synaptic hyperexcitability could contribute to axonal and NMJ degeneration in CMT.

#### Heat shock protein family Bs (HSPBs)

Small heat shock proteins, particularly HSPB1 (CMT2F) and HSPB8 (CMT2L), are linked to axonal CMT and dHMN through impaired proteostasis, cytoskeletal instability and mitochondrial dysfunction (Frasquet et al. 2021; Sarparanta et al. 2020). Direct mammalian NMJ evidence remains limited and highly context-dependent. Neuron-specific overexpression of mutant *HSPB1* (p.S135F, p.P182L) in mice causes axonal transport defects, progressive distal muscle denervation and NMJ loss without demyelination, supporting cell-autonomous toxicity (d’Ydewalle et al. 2011; Kim et al. 2016). In contrast, knock-in mice expressing human mutant *HSPB1* at lower, endogenous levels show minimal to no NMJ pathology even at advanced ages, emphasising strong dosage and model dependence (Bouhy et al. 2016).

For HSPB8, *Drosophila* mutant models demonstrate NMJ aggregation, reduced bouton number and impaired motor behaviour (Jabłońska et al. 2018). Overall, evidence for NMJ involvement in HSPB-related neuropathies is sparse and remains secondary to studies focused on axonal and mitochondrial pathology (Bouhy et al. 2016). However, the convergence of distal axon vulnerability, muscle-nerve interface destabilisation and stress-response dysregulation positions HSPBs as compelling candidates for further targeted mammalian NMJ investigation, particularly as heat shock proteins are known to aggregate at NMJs (Claeys et al. 2010; La Padula et al. 2016; Luo et al. 2008) and are increasingly recognised as key regulators of skeletal muscle development, maintenance and regeneration in neuromuscular disorders (Pomella et al. 2023; Sarparanta et al. 2020; Züchner & Vance 2006).

#### LMNA

More substantial evidence for NMJ dysfunction at the muscle interface comes from CMT-causative genes in contexts of dystrophy, ALS and myopathy. *LMNA* encodes lamin A/C, which are nuclear scaffold proteins that maintain connection between the nucleoskeleton and cytoskeleton (Castellano et al. 2025; Iyer et al. 2021). Diverse diseases termed laminopathies caused by *LMNA* mutation include recessive axonal CMT2B1, Emery-Dreifuss muscular dystrophy (EDMD), limb-girdle, and congenital muscular dystrophies. *LMNA* null mice exhibit hallmarks of axonopathy, as mirrored in human CMT2B1 phenotypes, though more classically involve muscle-centric pathology (De Sandre-Giovannoli et al. 2002; Ganassi & Zammit 2022).

Muscle-specific *LMNA* loss in EDMD is sufficient to disrupt NMJ formation, maintenance and function, producing early postsynaptic fragmentation, mislocalised synaptic nuclei, and rapsyn-mediated postsynaptic destabilisation. Aberrant innervation patterns, denervation-like transcriptional changes and synaptic transmission failure preceding overt myopathy are also observed upon muscle-specific deletion, while conditional deletion in motor neurons had no such impact (Gao et al. 2020; Méjat et al. 2009).

Other investigations into *LMNA* function demonstrate innovative experimental approaches to NMJ interrogation, including the use of puromycin SunSET labelling to directly quantify defective protein synthesis in mouse muscles, and iPSC-derived 3D platforms created to assess muscle-specific contribution to disease models (Maffioletti et al. 2018; Owens et al. 2020).

#### CHCHD10

These findings are paralleled by work on the gene *CHCHD10*, encoding a mitochondrial protein, where mutations are associated with ALS/FTD, axonal CMT2 and spinal muscular atrophy Jokela type (SMAJ) (Auranen et al. 2015; Genin et al. 2023, 2024). Knock-out and knock-in *CHCHD10*^*S59L/*+^ models of ALS show muscle-specific loss of *CHCHD10* is sufficient to impair NMJ integrity, and that mitochondrial dysfunction precedes NMJ degeneration, which is subsequently followed by motor neuron loss. Disrupted postsynaptic organisation is also observed via progressive endplate fragmentation and altered agrin-dependent AChR clustering (Anderson et al. 2019; Genin et al. 2019; Y. Xiao et al. 2020). Mice harbouring the *CHCHD10*^*G66V*^ SMAJ variant in both heterozygosity and homozygosity display no loss of motor neurons, reduction in NCVs or NMJ denervation, but possess reduced AChR areas in gastrocnemius muscle with advanced age (Harjuhaahto et al. 2025).

These models do not recapitulate CMT phenotypes, and the pathogenic roles of the encoded proteins likely differ from those in axonal CMT2 due to distinct variants and potential tissue-specific effects. Nevertheless, they reveal highly informative, distinct paradigms at the nerve-muscle interface such as: demonstrating that muscle-intrinsic dysfunction can destabilise NMJs independently of motor neurons; implicating postsynaptic structural proteins (rapsyn and agrin) as key determinants of NMJ instability; showcasing muscle-focused experimental approaches or early metabolic compromise; and revealing that primary neurotransmission defects can precede overt myopathy – cumulatively underscoring distinct mechanisms and early functional assessment as a sensitive and important readout of NMJ dysfunction in disease.

#### DNM2

Dynamin 2 (*DNM2*) provides an instructive counterexample that underscores the need for caution when extrapolating NMJ mechanisms across neuromuscular diseases. *DNM2* encodes a ubiquitous GTPase required for actin-membrane coupling, and endocytic vesicle trafficking across muscle, Schwann cells and neurons, and is also directly implicated in NMJ development via early postsynaptic enrichment (Lin et al. 2020; Proszynski et al. 2009; Sidiropoulos et al. 2012; Tinelli et al. 2013). Distinct heterozygous *DNM2* mutations cause either DI-CMT or centronuclear myopathy (CNM) (Gibbs et al. 2013; Massana Muñoz et al. 2019).

CNM models exhibit robust muscle-intrinsic NMJ pathology, including postsynaptic fragmentation, impaired AChR clustering, disorganised excitation–contraction coupling machinery, and mitochondrial abnormalities (Gibbs et al. 2013; Muñoz et al. 2020; Tinelli et al. 2013). In contrast, mice modelling CMT-associated *DNM2* mutations show absent or only mild NMJ phenotypes, with pathology instead centred on Schwann cell endocytosis and myelination defects (Massana Muñoz et al. 2019; Sidiropoulos et al. 2012). CMT-causing variants cluster in lipid-interacting domains, supporting alternative mechanisms distinct from the muscle-centric toxicity observed in CNM (Gómez-Oca et al. 2022).

Collectively, these comparative models demonstrate that while profound NMJ pathology can arise from muscle-intrinsic nuclear, mitochondrial, or membrane-trafficking defects, such mechanisms cannot be assumed to operate uniformly in CMT. Instead, they emphasise the need for prioritising disease- and tissue-specific interrogation of NMJs, while providing valuable conceptual frameworks for understanding synaptic vulnerability across neuromuscular disorders.

## Discussion

### Mouse model validity

Despite their widespread use, mouse models show substantial limitations in recapitulating NMJ pathology in CMT. Many genetically authentic mutant models fail to produce robust CMT phenotypes (including dominant *MFN2* mutations, *NEFL*-null, *GDAP1*-null, *TRIM2*-null, *HINT1*-null, *LSRAM1* and *DNM2* models) (Balastik et al. 2008; Barneo-Muñoz et al. 2015; Bogdanik et al. 2013; Jackson et al. 2012; Niemann et al. 2014; Pereira et al. 2020; Zhu et al. 1997); while others are constrained by embryonic or early postnatal lethality (e.g. *KIF5A*, *EGR2*, *DNM2*) (H. Chen et al. 2003; Soustelle et al. 2023; Sztretye et al. 2020). Genetic background further modulates phenotype severity, as is demonstrated when modelling CMT2D, complicating cross-study comparisons and reinforcing the need for careful model selection (Achilli et al. 2009; Fisher & Bannerman 2019; Kalotay et al. 2023; Seburn et al. 2006). Adding to this complexity, several CMT genes are primarily modelled in the context of ALS or other neurodegenerative diseases (e.g. KIFs, *VCP*, *PLEKHG5*, *BICD2*) (Clemen et al. 2018; T.-N. Huang et al. 2024; Hutchings et al. 2024; Lüningschrör et al. 2017; Martinez Carrera et al. 2018; Rossor et al. 2020), while others lack NMJ-focused analysis despite clear clinical relevance (e.g. *HK1*, *TRPV4*, *AIFM1*, *REEP1*, *BSCL2*, *COX6A1*) (Burns et al. 2026; Pipis et al. 2019; Rossor et al. 2024).

Beyond rodents, invertebrate and non-mammalian models provide complementary insights for NMJ research in CMT – reviewed recently in (Jeong & Lee 2024; Kitani-Morii & Noto 2020; Korzeniowska née Wiweger et al. 2025; Morant et al. 2021; Soh et al. 2020). *Drosophila* and *C. elegans* enable high-throughput genetic and functional interrogation of CMT-associated mutations (Kitani-Morii & Noto 2020; Soh et al. 2020). However, the absence of Schwann cells, myelinated axons and an adaptive immune system fundamentally restricts their relevance for modelling demyelinating CMT or axon-Schwann cell interactions central to NMJ stability (Bhattacharya 2023). Zebrafish overcome several of these constraints, since peripheral axons are myelinated, muscle fibre types are conserved, and the peripheral nervous system is highly accessible for live imaging during development (D’Rozario et al. 2017; Korzeniowska née Wiweger et al. 2025; Y. Xiao et al. 2015). Nonetheless, gene duplication, polymorphism and orthologue ambiguity complicate experimentation and interpretation (Gasanov et al. 2021).

Notably, for several CMT genes, NMJ pathology has been explored primarily beyond rodent models. *SGPL1* and *SORD* deficiency produce indications of synaptic morphological abnormalities in *Drosophila* (Atkinson et al. 2017; Cortese et al. 2020; Kitani-Morii & Noto 2020), while *RAB7* mutants (CMT2B) reveal distal axonal transport and NMJ maintenance defects without overt NMJ degeneration in *Drosophila* or zebrafish models (Basargekar et al. 2020; Cherry et al. 2013; Janssens et al. 2014; Wong et al. 2023). Jointly, these studies indicate that synaptic vulnerability may be detectable outside rodent systems, but require further assessment in mammalian models to establish conservation and functional significance – especially since the fly neuromuscular synapse relies on glutamate, rather than acetylcholine, for neurotransmission.

Across species, critical translational challenges also arise from fundamental differences between mouse and human NMJs. Human NMJs are smaller, more fragmented, possess thinner pre-terminal axons, deeper postsynaptic folds, and operate with a lower safety factor than mouse NMJs (Jones et al. 2017; Slater 2017). While murine NMJs exhibit pronounced age-related remodelling, human NMJs appear remarkably stable across adulthood, even in advanced age, injury, and cachexia (Boehm et al. 2020; Gupta et al. 2020; Jones et al. 2017). These discrepancies raise the risk that morphological degeneration observed in mice may not directly translate to functional failure in humans, or vice versa (Cahalan et al. 2022). Consequently, rather than thinking of mice as being able to model all neuromuscular aspects of a disease, it is more appropriate to harness them for understanding the proteins, pathways and processes that underpin pathology at the NMJ.

Large mammalian models may partially bridge this gap. Comparative studies demonstrate that sheep and pigs possess NMJ morphologies more similar than rodents to humans, supporting their translational value (Boehm et al. 2020). Naturally occurring inherited neuropathies in dogs or cows also provide physiologically relevant CMT models, though cost, ethics, and feasibility limit widespread adoption (Dittmer et al. 2022; Korzeniowska née Wiweger et al. 2025; Skedsmo et al. 2021). More recently, in vitro modelling has made substantial progress in CMT research and is increasingly recognised as a relevant, complementary approach for interrogating NMJ pathology. Human iPSC-derived systems are particularly valuable because they permit reconstruction of contractile neuromuscular contacts, while allowing direct assessment of mutation-induced pathology using isogenic controls and reducing confounds from species-specific differences and genetic backgrounds (Van Lent et al. 2024; K. Zhang et al. 2021b). The rapid proliferation of recent reviews on iPSC-based neuropathy models, neuromuscular organoids, assembloids and bioengineered nerve-muscle “on-a-chip” constructs reflects the growing consensus that NMJ-centric approaches are critical for understanding distal axonal failure in CMT (Castellanos-Montiel et al. 2021; Cho & Jang 2021; Scherrer et al. 2025; Van Lent et al. 2024; Yang et al. 2025). 3D CMT organoid models have exemplified the capacity for in vitro myelin production, and demonstrated that downregulation of *PMP22* ameliorates myelin abnormalities – validating the relevance of these systems for mechanistic and therapeutic studies in CMT1A (Van Lent et al. 2023). However, organoids lack vascularisation and directional growth, and typify existing caveats of in vitro modelling, such as restricted scalability, lack of standardised differentiation protocols, variable maturation, and extensive technical complexity of platform assembly (Bombieri et al. 2024; Castillo Bautista & Sterneckert 2023; Van Lent et al. 2024).

Altogether, while physiological and molecular differences between species and platforms represent challenges, the most informative strategies for CMT NMJ research will likely involve integrated, multi-platform approaches. These should include in vitro systems to dissect early synaptic mechanisms and human-specific pathology, as well as animal and especially mouse models, which remain essential due to their experimental accessibility and genetic tractability for validating circuit-level and organism-level outcomes.

### Current limitations

Although NMJ involvement in CMT is now better supported conceptually, the evidence base and approach to interrogation remains uneven, and the breadth of methods for investigation conventionally employed from the possible toolkit remains restricted. Several limitations constrain the strength and comparability of findings generated from the growing use of mice for investigating NMJ pathology in CMT. A recurring issue is small or inappropriate sample sizes, which is particularly problematic due to intrinsic NMJ heterogeneity. Morphology varies markedly between individual NMJs, across developmental stages, and even within single muscles (Mech et al. 2020). Accordingly, adequate sampling is essential to capture biologically meaningful patterns. However, the reported approaches in CMT studies reviewed here vary widely, ranging from one to three NMJs in one to two animals, to hundreds of NMJs per animal, thus foregrounding a spectrum from underpowered analyses to inefficient oversampling. Critically, the experimental unit in NMJ studies should be the animal, not the individual synapse; treating NMJs as independent data points introduces pseudoreplication, inflates significance and violates statistical assumptions.

These issues align with broader concerns in animal experimental design. Robust inference depends on correct identification of experimental units, a priori sample size calculations, randomisation, blinding and transparent exclusion criteria. Yet these practices remain inconsistently reported in NMJ studies and in vivo research more broadly (Lazic 2010; Macleod et al. 2015; Wilson et al. 2023). In a field already complicated by extensive model heterogeneity, underpowered designs increase the risk of false-positive and irreproducible conclusions.

Methodological limitations further complicate interpretation. Most NMJ analyses rely on immunostained fixed tissue, where technical variability, antibody selection, batch effects and staining quality can compromise robustness or comparability. Although automated platforms have improved objectivity, many omit Schwann cell morphology as a core NMJ component and are often applied to 2D projections that conceal full, 3D organisation patterns. Uptake of quantitative tools moreover remains inconsistent, and the parallel development of different platforms duplicated in name for different species (e.g. NMJ-Analyzer) (Mejia Maza et al. 2021; Singh et al. 2023) highlights limited standardisation, field-wide coordination and uptake. A major challenge is the lack of consensus in defining, quantifying and interpreting structural denervation, perhaps the most frequently assessed NMJ metric. Some studies use a single overlap threshold between pre and postsynaptic markers (e.g. 50%) (Nandini et al. 2019; J. R. Nicks et al. 2013), which collapses a continuum of synaptic occupancy into binary categories, allowing markedly under-innervated endplates to be classified as intact, thereby obscuring biologically relevant gradients of denervation. Alternate studies or approaches distinguish partial from complete denervation in distinct categories and employ more stratified percentage cut-offs based on visual assessment (Simkin et al. 2025; Sleigh et al. 2014b), or with these categories defined only by qualitative states not percentages (Pérez-López et al. 2025; Ricardez Hernandez et al. 2025). Across the field, these criteria are not consistently reported and are often acquired from variable contexts, such as 2D flattened projections, 3D volumetric reconstructions or manual assessment of muscles down the eyepieces, using microscope objectives ranging from 20 to 100 × magnifications. These factors render denervation rates highly difficult to compare between studies. Moreover, partial denervation spectra do not necessarily provide instructive information about functional compromise, with NMJs being able to maintain effective transmission despite pronounced morphological abnormalities, due to the neuromuscular safety factor (Gillingwater et al. 2002; Kong et al. 2009; Nagel et al. 1990). Furthermore, reporting of innervation/denervation percentages alone, without quantifying the total number of NMJs per muscle (Fig. 1D), can lead to an underestimation of the extent of pathology, since denervated synapses with time become lost and undetectable by immunohistochemistry (Comley et al. 2022).

Overreliance on static morphology and visual classification therefore risks misinterpretation. Structural changes may reflect regeneration or plasticity rather than degeneration, while apparently intact synapses may be functionally compromised through mechanisms such as conduction block (Baloh et al. 2009). Thin tissue sections and muscle fibre teasing exacerbate these issues by preventing reconstruction of full axonal trajectories (Fig. 1B) and assessment of total NMJ numbers, increasing processing artefacts and obscuring motor unit-level patterns. Initial choices of muscle made for analysis can additionally shape ultimate interpretations: while thin, flat muscles facilitate wholemount analysis, they may not reflect disease-relevant pathology unless carefully selected (Sleigh et al. 2020; Sleigh et al. 2014b; Spaulding et al. 2016). Internal muscles such as the diaphragm or triangularis sterni may be biologically appropriate in some models, for instance those subtypes with respiratory involvement (S. E. Holbrook et al. 2024; Torres et al. 2025; Villalón et al. 2018), but remain less so in models and diseases with distal limb-restricted phenotypes (Court et al. 2008a; Patzkó et al. 2012). As further exemplification, investigation in ALS frequently identifies fast-twitch distal muscles as being more strongly susceptible to early NMJ disruption than slow-twitch muscles (Alhindi et al. 2022; Frey et al. 2000; Vinsant et al. 2013). Reliable identification of axonal sprouting and reinnervation dynamics within muscles moreover requires additional markers, such as GAP43 or Schwann cell-associated proteins, or longitudinal in vivo imaging, rather than inference from static axonal morphology alone (Gomez-Sanchez et al. 2017; J. R. Nicks et al. 2013). Since CMT drives selective, distal dysfunction, it is also evident that principally studying axons at more proximal locations, for instance in the commonly assessed sciatic nerve, can result in underestimation of pathology. Indeed, the peripheral synaptic degeneration without loss of motor neurons observed in many CMT mouse models suggests that there is even selectivity within the motor unit, highlighting the utility of selective focus on NMJ investigation.

Importantly, functional assessment of transmission remains underused, despite evidence across multiple CMT models that NMJs are an early and sensitive site of physiological dysfunction when examined (Bowerman et al. 2017; Court et al. 2008a; Grønnebæk et al. 2024; Scurry et al. 2016; Spaulding et al. 2016), and should be combined with morphological analyses for a more accurate picture of NMJ integrity.

Finally, there is a paucity of longitudinal studies tracking NMJ maturation, degeneration and attempted repair over time, across muscles and between models. This contrasts with contexts beyond CMT, such as tracking deterioration over time in sarcopenia, pro-regenerative responses seen after both acute and chronic denervation, and NMJ restoration following endurance training (Bakooshli et al. 2023; Sarto et al. 2024; Yamaguchi et al. 2025). Within CMT, this gap makes it challenging to distinguish early pathogenic changes from secondary degeneration or compensatory remodelling – matters of particular importance given the intrinsic plasticity of the neuromuscular system and the numerous, diverse possible manifestations of pathology detected at the NMJ.

### Therapeutic interventions

There is currently no curative therapy for CMT, and clinical strategies remain largely supportive, focusing on symptom and/or pain management (Burns et al. 2026; Pareyson & Marchesi 2009). The absence of disease-modifying treatments reflects the substantial genetic and mechanistic heterogeneity of CMT, which hinders target prioritisation and limits the success of single-pathway approaches (Juneja et al. 2019). Despite extensive preclinical efforts, relatively few therapeutic approaches have progressed to clinical trials, and none have yet achieved regulatory approval (Vendredy et al. 2025). Gene-based therapies increasingly target causal mutations in CMT, including via gene replacement for recessive forms and allele-specific suppression of dominant genes, such as *PMP22* and *GARS1* (Gautier et al. 2021; Morelli et al. 2019; Stavrou et al. 2023). Comprehensive discussion of therapeutic strategies in CMT have been reviewed recently (Bosco et al. 2021; Fridman & Saporta 2021; Pisciotta & Pareyson 2023; Stavrou et al. ).

Axonal degeneration is a major destabiliser of NMJ integrity and a primary or secondary driver of pathology across both axonal and demyelinating forms of CMT, making it an attractive therapeutic focus. While advances in *HDAC6* inhibition have been discussed elsewhere (Markworth et al. 2021), recent attention has centred on targeting programmed axon degeneration pathways, particularly the Wallerian degeneration cascade, for its high remedial potential in CMT (Moss & Höke 2020; Stavrou et al. 2023).

In non-genetic neuropathies, inhibition of *SARM1* confers robust neuroprotection, including in mechanical axotomy, chemotherapy-induced neuropathy and metabolic dyslipidaemia (Geisler et al. 2016; Osterloh et al. 2012; Turkiew et al. 2017). Translation to inherited neuropathies, however, has so far yielded mixed outcomes. In rats, modulation of the Wallerian degeneration pathway partially preserved axon integrity and NMJ structure in *PMP22* CMT1A and *MFN2*^*H361Y*^ CMT2A models (Meyer Zu Horste et al. 2011; Sato-Yamada et al. 2022). In *MPZ*^*T124M*^ CMT2J mice, *SARM1* deletion reduced axonal degeneration and preserved motor nerve integrity, but failed to correct electrophysiological deficits, indicating selective and incomplete protection (Claessens 2025).

In contrast, removal of SARM1 has little to no benefit in most other rodent CMT models. Genetic *SARM1* depletion did not improve neuromuscular phenotypes in the C3-PMP CMT1A model (Moss et al. 2022) or following neonatal AAV9-mediated inhibition in *Gars*^*ΔETAQ*^ (CMT2D), *NEFL*^*N98S*^ (CMT2E) and *IGHMBP2*^*Y918C*^ (CMT2S) mice (Rice et al. 2025). The *Wlds* mutation also failed to rescue NMJ innervation or axonal pathology in *Gars*^*P278KY/*+^ mice (Stum et al. 2011) and *SARM1* depletion was ineffective in models of *GJB1* (CMT1X), *FIG4* (CMT4J) and *KIF1A*-associated neuropathy (Hatton et al. 2025).

Collectively, these findings indicate that programmed axon degeneration can contribute to NMJ destabilisation and neuropathy in CMT, but in a context-, mutation- and potentially species-dependent manner. While selected CMT subtypes may benefit from *SARM1* inhibition, this pathway is unlikely to represent the universal driver of pathology or a single therapeutic target. Defining when and where *SARM1* is first heterogeneously activated across specific genotypes will be essential for predicting future therapeutic benefit for axonal, NMJ and clinical outcomes in CMT.

More explicitly, the NMJ is now an established therapeutic target in a range of neuromuscular and myasthenic disorders – including through strategies that modulate presynaptic neurotransmitter release, preserve postsynaptic excitability, enhance agrin-LRP4-MuSK signalling, or reduce inhibitory postsynaptic currents to improve neuromuscular transmission (Alhindi et al. 2022; DeHart-McCoyle et al. 2023; Moss et al. 2025; Qaisar 2023). Consistent with accumulating evidence that the NMJ plays a pivotal role in CMT, there is increasing recognition of the NMJ as a direct pathological site and viable therapeutic target in CMT itself (Pisciotta & Pareyson 2023; Scherrer et al. 2025; Soh et al. 2020).

Acetylcholinesterase inhibition with physostigmine improves neuromuscular transmission and NMJ function in CMT2D mice (Spaulding et al. 2016), demonstrating the proof-of-principle that augmenting synaptic efficacy can ameliorate downstream dysfunction. However, translation to patients has so far been mixed: the beta-2 adrenergic receptor agonist salbutamol is an effective treatment strategy for patients with congenital myasthenic syndromes, and can rescue both muscle weakness along with defective NMJ structure (M. Lee et al. 2018; Webster et al. 2020), but only improved general fatigue and not specific muscle strength in a small trial of dHMN patients that included CMT2D and CMT2M (McMacken et al. 2023).

More recently, targeting of the skeletal muscle chloride channel ClC-1 has emerged as a particularly promising strategy. ClC-1 inhibition with the small molecule NMD670 clinically restores neuromuscular transmission and muscle function in MG patients (Skov et al. 2024) and improves muscle contractility in mouse models of CMT1A and CMT2D (Grønnebæk et al. 2024). Importantly, clinical studies demonstrate that NMJ transmission deficits are present across CMT genotypes, and a Phase 2a trial of NMD670 in CMT patients is now underway (Grønnebæk et al. 2024; NMD Pharma 2025) (trial ID: NCT06482437). Together, these findings support the NMJ as a therapeutically actionable site in CMT with genuine translational potential.

### Axonal versus demyelinating CMT: convergent vulnerability, distinct routes

Across the broad genetic spectrum of CMT, NMJ involvement emerges as a recurrent but subtype-dependent feature (Fig. 2C, Supplementary Table 1), revealing both partially shared, and sometimes primary, synaptic vulnerability, as well as marked divergence in how neuromuscular connectivity fails. A subset of axonal CMT genes implicate the NMJ as a key site of distal dysfunction reflective of the unique demands of long motor axons, but with significant heterogeneity in mechanistic resolution. NMJ pathology when directly assessed often appears length-dependent and progressive. Mutant *GARS1* models provide the clearest causal framework, while work on *IGHMBP2* demonstrates CMT-specific NMJ involvement – capable of distinguishing CMT2S from SMARD1– yet for most genes, conclusions remain otherwise inferred from indirect or static measures.

By contrast, demyelinating CMT genes more consistently implicate the NMJ as a site of secondary destabilisation driven by Schwann cell dysfunction, occasionally preceding or occurring without overt axonal loss. Across *PMP22*-, *MPZ*-, and *EGR2*-related models, impaired conduction, preterminal demyelination, and metabolic insufficiency promote functional denervation with attempted but unsuccessful reinnervation. Notably, these features distinguish nuances in demyelinating CMT from classical, dying-back axonopathies and suggest repeated cyclical progressions of destabilisation and ineffective repair at the synapse in contrast to linear degenerative trajectories.

Despite these differences, both axonal and demyelinating forms of CMT converge on the NMJ as a critical control site for motor output, with unified patterns of functional denervation and impaired transmission possible at times even in spite of preserved axonal continuity. Collective dysfunction can arise through intersecting mechanisms including chronic disruption of axon maintenance and Schwann cell support or, more uniquely, direct neomorphic perturbation of synaptic signalling at the NMJ itself. Accordingly, NMJ pathology in CMT is not confined to secondary synaptic compromise downstream of neuronal stress, but can also represent early and, at times, autonomously targeted sites of dysfunction, through which diverse pathogenic routes can impair synaptic transmission, maintenance or regeneration to drive clinical neuropathy. As genetic sequencing advances increasingly blur boundaries between CMT subtypes, defining such convergent features becomes ever more important for interpreting shared mechanisms of motor impairment.

### Future directions

Addressing outstanding gaps in CMT NMJ research is not trivial in part because of the sheer number of CMT models, genetic causes and implicated mechanisms (Bosco et al. 2021; Juneja et al. 2019). Despite these challenges, however, CMT is uniquely positioned to advance NMJ biology precisely *because* its genetic diversity spans axon and Schwann cell mechanisms within an intelligible clinical spectrum converging at a distinct physical nexus. Accordingly, with improved standardisation, rigorous experimental design and broader incorporation of functional, appropriately regionalised and longitudinal approaches, NMJ-focused studies are well positioned to deliver decisive mechanistic insight into the selective peripheral neuropathogenesis of CMT (Scherrer et al. 2025).

Recent advances in single-nucleus, spatial, and multi-omics profiling (Amoretti et al. 2025; Ham et al. 2025; Negro et al. 2022) now render the complex multicellular interface of the NMJ molecularly tractable at resolutions previously inaccessible due to difficulties of peripheral synapse isolation (Hindley et al. 2023). Applied longitudinally to disease models (Laszlo et al. 2022; Piol et al. 2023), these approaches present a powerful framework to sublimate understanding of how cell type-specific transcriptional states and local translational programmes at individual NMJs evolve throughout disease and converge or differ across CMT subtypes to ultimately drive synaptic failure and neuropathy.

Therapeutically, accumulating evidence in mouse models indicates it is possible to restore NMJ function after the emergence of pathology, while demonstrating that preservation of axons alone is insufficient to maintain neuromuscular function in many CMT contexts. Instead, stabilising or augmenting NMJ transmission represents a complementary and increasingly actionable strategy, as indicated by emerging NMJ-targeted interventions. As such, NMJ pathology in CMT provides not only mechanistic insight into distal neurodegeneration, but a translational framework for preserving motor output across genetically diverse neuropathies.

## Supplementary Information

Below is the link to the electronic supplementary material.

Supplementary file1 (DOCX 151 KB)

## Data Availability

No datasets were generated or analysed during the current study.
