# Supplementary material for: Junctions in Jeopardy: the neuromuscular junction is a selective pathological target in Charcot-Marie-Tooth disease
Source: Mamm Genome. 2026 May 22;37(1):72. doi: 10.1007/s00335-026-10238-z (PMC13197389; doi:10.1007/s00335-026-10238-z)
Supplement: Supplementary file 1 — Supplementary file1 (DOCX 151 KB) [file 335_2026_10238_MOESM1_ESM.docx]

**Supplementary Table 1:** A summary of Fig. 2C, delineating types of pathology observed at the NMJ mapped to canonical CMT and CMT-associated gene variants and models. Information is organised by the primary site of action (1. axonal / presynaptic, 2. pre and / or postsynaptic, and 3. postsynaptic).

| ***Feature of NMJ Pathology Observed*** | ***Gene and Pathogenic Variant or Associated Condition*** | | ***References*** |
| --- | --- | --- | --- |
| **Axonal / Presynaptic** |  | |  |
| Functional Denervation / Conduction Block | *MPZ* (P_0_ ^tg^) | | (Yin et al., 2004) |
|  | *EGR2^I268N^*, MPZ-cre-*EGR2-*cKO | | (Baloh et al., 2009; Zotter, 2020) |
|  | *SH3TC2^-/-^* | | (Morelli et al., 2017) |
|  | *PRX^⁻/⁻^* | | (Court, Brophy, et al., 2008) |
| Structural Denervation (Axonal) | *GARS1^P278KY^*, *^C201R^* and *^ΔETAQ^* | | (Achilli et al., 2009; Benoy et al., 2018; W. He et al., 2015; Mejia Maza et al., 2021; Morelli et al., 2019; Seburn et al., 2006; Sleigh, Burgess, et al., 2014; Sleigh et al., 2020) |
|  | *NEFL^E397K^* | | (Pérez-López et al., 2025) |
|  | *DYNC1H1^H304R^* | | (Nandini et al., 2019; Sabblah et al., 2018) |
|  | *GDAP1^-/-^* | | (Barneo-Muñoz et al., 2015; Civera-Tregón et al., 2021; Fernandez-Lizarbe et al., 2019) |
|  | *MFN2^R94Q^ and ^H361Y^* | | (Sato-Yamada et al., 2022; Y. Zhou et al., 2019) |
|  | *IGHMBP2* *^ΔE365^ and ^Y918C^* | | (Martin et al., 2022; Ricardez Hernandez et al., 2025) |
|  | *HSPB1^P182L^* and *^S135F^* | | (d’Ydewalle et al., 2011; Kim et al., 2016) |
|  | *LMNA ^-/-^, ^H222P^* and HSA-cKO | | (De Sandre-Giovannoli et al., 2002; Gao et al., 2020; Méjat et al., 2009) |
|  | *CHCHD10* (ALS) | | (Anderson et al., 2019; Genin et al., 2023; Y. Xiao et al., 2020) |
| Structural Denervation (Demyelinating) | *PMP22* (CMT1A: *PMP*-C22 and C3) | | (Huxley et al., 1996; Meyer Zu Horste et al., 2011; Moss et al., 2022; Robertson et al., 2002; Sereda et al., 1996) |
|  | *PMP22* (CMT1E: Tr-J*)* | | (Gale et al., 1982; J. R. Nicks et al., 2013) |
|  | *MPZ* (P_0_ ^tg^) | | (Yin et al., 2004) |
|  | *SH3TC2^-/-^* | | (Morelli et al., 2017) |
|  | *MPZ-*cre*-FGD4-*cKO | | (El-Bazzal et al., 2023) |
|  | *GJB1^-/-^* and *^R75W^* | | (Groh et al., 2010; Kaur et al., 2023; Klein et al., 2015; Lang et al., 2025; Scherer et al., 1998) |
|  | *CNTNAP1^-/-^* | | (Saifetiarova et al., 2017) |
| Sprouting or Attempted Regeneration | *PMP22* (CMT1A: ^Tg^) | | (Ang et al., 2010) |
|  | *PMP22* (CMT1E: Tr-J*)* | | (Gale et al., 1982; J. R. Nicks et al., 2013) |
|  | *MPZ* (P_0_ ^tg^) and *^T124M^* | | (Shackleford et al., 2022; Yin et al., 2004) |
|  | *EGR2^I268N^* | | (Baloh et al., 2009) |
|  | *IGHMBP2* (SMARD1: nmd^2J^) | | (Grohmann et al., 2004) |
| Maturation Deficits | *GARS1^C201R^* and ^P278KY^ | | (Sleigh, Grice, et al., 2014) |
|  | *DYNC1H1^H304R^* | | (Sabblah et al., 2018) |
|  | *SLC25A46 (atc)* | | (Terzenidou et al., 2017) |
|  | *PMP22 (Tr-J)* | | (Scurry et al., 2016) |
| Nodal Disruption | *MPZ^T124M^* | | (Shackleford et al., 2022) |
|  | *CNTNAP1^-/-^* | | (Chang et al., 2023; Saifetiarova et al., 2017) |
|  | *SH3TC2^-/-^* | | (Morelli et al., 2017) |
|  | *LITAF^W116G^* | | (S. M. Lee et al., 2013) |
| Cytoskeletal Instability | *GARS1^P278KY^* and *^C201R^* | | (Benoy et al., 2018; Mo et al., 2018) |
|  | *YARS1^E196K^* | | (Ermanoska et al., 2023; Morant et al., 2021) |
|  | *NEFL ^N98S^, ^E396/397K^ and ^Q332/333P^* | | (Perez-Olle et al., 2004; Stone et al., 2019, 2021) |
|  | *MFN2^R94Q^* | | (Picci et al., 2020) |
|  | *HSPB1^P182L^* and *^S135F^* | | (d’Ydewalle et al., 2011; Kim et al., 2016) |
|  | *CNTNAP1^-/-^* | | (Saifetiarova et al., 2017) |
| Axonal Transport Deficits | **Signalling endosomes:** | *GARS1^C201R^* and *^ΔETAQ^* | (Sleigh et al., 2023) |
|  |  | *YARS1^E196K^* | (Rhymes et al., 2024) |
|  |  | *MPZ^T124M^* | (Claessens, 2025) |
|  | **Mitochondria:** | *GDAP^-/-^* | (Niemann et al., 2014) |
|  |  | *GARS1^C201R^* | (Benoy et al., 2018) |
|  |  | *NEFL^P22S^, NEFL^N98S^* | (Dequen et al., 2010; M. A. Saporta et al., 2015) |
|  |  | *HSPB1^P182L^* and *^S135F^* | (d’Ydewalle et al., 2011; Kim et al., 2016) |
|  |  | *RAB^V162M^* | (Wong et al., 2023) |
|  |  | *PDK3^R158H^* | (Perez-Siles et al., 2020) |
| Schwann Cell Proteostatic Stress or Accumulation | *PMP22* (CMT1A: ^Tg^) | | (Fledrich et al., 2014) |
|  | *PMP22* (CMT1E: Tr-J) | | (D’Urso et al., 1998; Okamoto et al., 2013; Tobler et al., 2002) |
|  | *MPZ^R98C^* and *^S63del^* | | (Pennuto et al., 2008; M. A. C. Saporta et al., 2012; Wrabetz et al., 2006) |
|  | *GJB1* (Many missense variants) | | (Chu et al., 2022; Kleopa et al., 2012) |
| **Pre and / or Postsynaptic** |  | |  |
| Morphological Alterations | *GARS1^P278KY^Y and ^C201R^* | | (Achilli et al., 2009; Mejia Maza et al., 2021; Seburn et al., 2006; Sleigh et al., 2020; Sleigh, Grice, et al., 2014) |
|  | *DYNC1H1^H304R^* | | (Nandini et al., 2019; Sabblah et al., 2018) |
|  | *MFN2^H361Y^* | | (Sato-Yamada et al., 2022) |
|  | *PMP22* (Tr-J) | | (Scurry et al., 2016) |
|  | *MPZ* (P_0_ ^tg^) and *^R98C^* | | (Patzkó et al., 2012; Yin et al., 2004) |
|  | *MPZ-c*re*-EGR2-*cKO | | (Zotter, 2020) |
|  | GJB1*^-/-^* | | (Kagiava et al., 2019; Kaur et al., 2023) |
|  | *PRX^-/-^* | | (Court, Brophy, et al., 2008) |
|  | *SH3TC2^-/-^* | | (Cipriani et al., 2018) |
|  | *CNTNAP1^-/^* | | (Saifetiarova et al., 2017) |
|  | *LMNA ^-/-^, ^H222P^* and HSA-cKO | | (Gao et al., 2020; Méjat et al., 2009) |
|  | *DNM2* (CNM) | | (Muñoz et al., 2020; Tinelli et al., 2013) |
|  | *SYT2* (CMS) | | (Bauché et al., 2020) |
|  | *CHCHD10* (ALS and SMAJ) | | (Anderson et al., 2019; Genin et al., 2019; Harjuhaahto et al., 2025; Y. Xiao et al., 2020) |
| Neurotransmission Disruption | *GARS1^P278KY^* and *^C201R^* | | (Spaulding et al., 2016) |
|  | *PMP22* (Tr-J) | | (Meekins et al., 2007; Scurry et al., 2016) |
|  | *MPZ* (P_0_ ^tg^) | | (Yin et al., 2004) |
|  | *NEFL^N98S^* | | (M. A. Saporta et al., 2015) |
|  | *MFN2^R364W^* | | (M. A. Saporta et al., 2015) |
|  | *IGHMBP2* (CMT2S) | | (Smieszek et al., 2025) |
|  | *SYT2* (CMS) | | (Fionda et al., 2021; McMacken et al., 2023; Pang et al., 2006) |
|  | *SLC12A6^-/-^* (Andermann syndrome) | | (Bowerman et al., 2017) |
| Mitochondrial Abnormalities | *GARS1^P278KY^* and *^C201R^* | | (Benoy et al., 2018; Spaulding et al., 2016) |
|  | *MFN2^T105M^* and *^L643P^* | | (Franco et al., 2020; Hines et al., 2023) |
|  | *GDAP1^-/-^* | | (Barneo-Muñoz et al., 2015; Civera-Tregón et al., 2021; Niemann et al., 2014) |
|  | *MPZ^T124M^* | | (Claessens, 2025) |
|  | *HSPB8^K141N^* | | (Bouhy et al., 2016) |
|  | *RAB^V162M^* | | (Wong et al., 2023) |
|  | *CNTNAP1^-/-^* | | (Saifetiarova et al., 2017) |
|  | *LITAF^W116G^* | | (S. M. Lee et al., 2013) |
|  | *DHTKD1^-/-^ and ^Y486^* | | (Luan et al., 2020; Xu et al., 2018) |
|  | *SLC25A46 (atc and KD)* | | (Ali et al., 2020; Suda et al., 2018; Terzenidou et al., 2017) |
|  | *PDK3^p.R158H^* | | (Kennerson et al., 2013; Narayanan et al., 2022) |
|  | *CHCHD10* (ALS) | | (Genin et al., 2019) |
|  | *DNM2* (CNM) | | (Tinelli et al., 2013) |
| Trophic Receptors and Neomorphic Interactions | **Mutant *GARS1:*** NRP1, TrkB, Plexins | | (Grice et al., 2018; W. He et al., 2015; Ozes et al., 2024; Sleigh, Dawes, et al., 2017; Sleigh et al., 2023; Sleigh, Gómez-Martín, et al., 2017) |
|  | **Mutant *YARS1:*** TrkB | | (Blocquel et al., 2017; Rhymes et al., 2024) |
| Aggregation / Accumulation | *GARS1^P278KY^* | | (Grice et al., 2015, 2018) |
|  | *NEFL^N98S^ and hNEFL^E397K^* | | (Adebola et al., 2015; Lancaster et al., 2018; Rice et al., 2025; Shen et al., 2011; Villalón et al., 2017) |
|  | HSPBs *(DNAJB2, HSPB3, HSP90β*) | | (Claeys et al., 2010; La Padula et al., 2016; Luo et al., 2008) |
|  | *DMN2 (CNM)* | | (Gibbs et al., 2013) |
| Translation Alterations | *GARS1^G240R^* and *^G526R^* | | (Mendonsa et al., 2021; Mora et al., 2025; Niehues et al., 2015) |
|  | *YARS^E196K^* | | (Bervoets et al., 2019; Niehues et al., 2015) |
|  | *IGHMBP2: ^D565N^ > ^H922Y^* | | (Vadla et al., 2024) |
| **Postsynaptic** |  | |  |
| Scaffolding Protein Disruption | HSA*-LMNA-cKO* | | (Gao et al., 2020) |
|  | HSA*-CHCHD10-cKO* | | (Y. Xiao et al., 2020) |
